# Elastic Buffering Layer on CuS Enabling High-Rate and Long-Life Sodium-Ion Storage

**DOI:** 10.1007/s40820-022-00924-3

**Published:** 2022-09-23

**Authors:** Yuanhua Xiao, Feng Yue, Ziqing Wen, Ya Shen, Dangcheng Su, Huazhang Guo, Xianhong Rui, Liming Zhou, Shaoming Fang, Yan Yu

**Affiliations:** 1 Key Laboratory of Surface and Interface Science and Technology, Zhengzhou University of Light Industry, Zhengzhou, 450002 People’s Republic of China; 2 Institute of Nanochemistry and Nanobiology, School of Environmental and Chemical Engineering, Shanghai University, Shanghai, 200444 People’s Republic of China; 3 Institute School of Materials and Energy, Guangdong University of Technology, Guangzhou, 510006 People’s Republic of China; 4 Hefei National Research Center for Physical Sciences at the Microscale, Department of Materials Science and Engineering, National Synchrotron Radiation Laboratory, CAS Key Laboratory of Materials for Energy Conversion, University of Science and Technology of China. Hefei, Anhui, 230026 People’s Republic of China

**Keywords:** CuS, Elastic buffering layer, Polyaniline, Long life, Sodium-ion batteries

## Abstract

The latest view suggests the inactive core, surface pulverization, and polysulfide shuttling effect of metal sulfides are responsible for their low capacity and poor cycling performance in sodium-ion batteries (SIBs). Whereas overcoming the above problems based on conventional nanoengineering is not efficient enough. In this work, erythrocyte-like CuS microspheres with an elastic buffering layer of ultrathin polyaniline (PANI) were synthesized through one-step self-assembly growth, followed by *in situ* polymerization of aniline. When CuS@PANI is used as anode electrode in SIBs, it delivers high capacity, ultrahigh rate capability (500 mAh g^−1^ at 0.1 A g^−1^, and 214.5 mAh g^−1^ at 40 A g^−1^), and superior cycling life of over 7500 cycles at 20 A g^−1^. A series of in/ex situ characterization techniques were applied to investigate the structural evolution and sodium-ion storage mechanism. The PANI swollen with electrolyte can stabilize solid electrolyte interface layer, benefit the ion transport/charge transfer at the PANI/electrolyte interface, and restrain the size growth of Cu particles in confined space. Moreover, finite element analyses and density functional simulations confirm that the PANI film effectively buffers the volume expansion, suppresses the surface pulverization, and traps the polysulfide.

**Supplementary Information:**

The online version contains supplementary material available at 10.1007/s40820-022-00924-3.

## Highlights

- Erythrocyte-like CuS microspheres were encapsulated in ultrathin polyaniline (PANI) layer coating.
- PANI swollen by electrolytes stabilizes solid electrolyte interface layer and benefits the ion transport and charge transfer at the PANI/electrolyte interface.
- Multi-functional PANI coating ensures an outstanding comprehensive performance for sodium-ion storage.

**Supplementary Information:**

The online version contains supplementary material available at 10.1007/s40820-022-00924-3.

## Graphical abstract

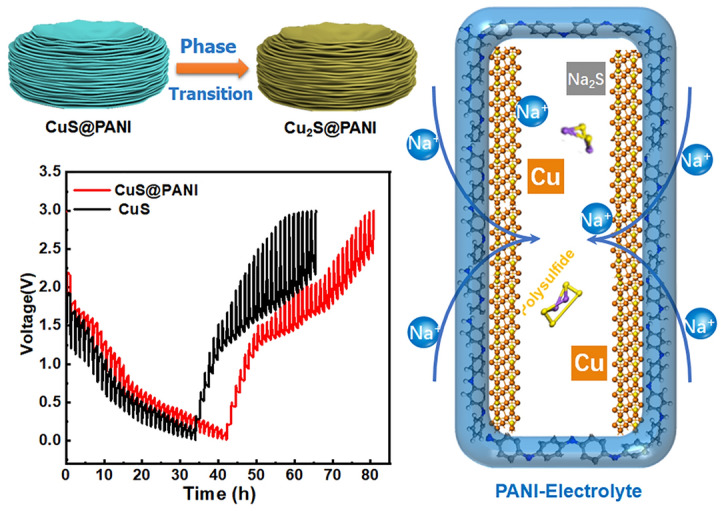

## Introduction

Along with the advance in the industry, commercial lithium-ion batteries (LIBs) may be unable to meet the rapidly increasing requirements in large-scale energy storage owing to the limited lithium resources [1, 2]. Accordingly, sodium-ion batteries (SIBs) with enriched sodium resources are emerging as one of the most promising alternatives to LIBs [3, 4]. Although lithium and sodium share similar properties as alkali metals, graphite, as the most common negative electrode of commercial LIBs, shows low reversible capacity (≈35 mAh g^−1^) in SIBs because Na^+^ embedded between graphite layers cannot form a stable NaC_*x*_ intercalation compound [5]. Afterward, it was found that the amorphous hard carbon shows enhanced Na-ion storage performance, but it still suffers from insufficient rate capacity, low initial Coulombic efficiency, and poor cycling stability [6–8]. Therefore, it is urgent to search for efficient anode materials for SIBs.

Metal sulfides (MSs) possess high theoretical capacities and fast reaction kinetics of phase transformation, which have been widely studied for high-performance SIBs [9, 10]. However, *in situ* real-time magnetic monitoring technology reveals the presence of "inactive core" in the interior caused by the sluggish kinetics of larger Na-ion radius and severe surface pulverization of MSs, results in its low capacity and poor cycling performance for SIBs [11]. Thus, MSs nanostructure with a short ion transmission path will facilitate Na^+^ insertion/extraction and highly utilize active materials. Furthermore, the pulverization caused by volume expansion during the typical conversion reaction usually requires the construction of hollow nanostructure and carbon-based composites [12]. However, the hollow nanostructure generally requires a complicated template-based strategy and has a low tap density (often < 0.7 g cm^−3^) [13]. Despite carbon-based composites proving high electronic conductivity, the low elongation of nanocarbon (e.g., < 10% for graphene) is not sufficient to buffer the more considerable volume expansion [14–16]. Differently, conductive polymer combines the advantages of high elongation and conductivity [17], which would bring a great chance to overcome the pulverization and low cyclability of MSs. Meanwhile, polymer coating displays a great potential to avoid the polysulfide shuttling issue in MSs anodes during the long-term cycling process.

Among the various MSs materials, copper sulfide (CuS) with layered structure has attracted much more attention because of its resource abundance and high capacity in SIBs [18]. However, the unique Cu aggregation behavior in CuS will promote the growth of Cu particles even to 38 nm in size during the electrochemical conversion reaction, which leads to huge local stress, strain, and particle cracking [19]. Therefore, coating the above-mentioned conductive polymer on the surface of CuS is an effective strategy for constructing high-performance SIBs anodes.

Herein, we report a facile solvothermal approach to prepare erythrocyte-like CuS microspheres (consisting of thin nanosheets) initially, and after an *in situ* polymerization, an ultrathin coating layer (~ 2.0 nm thickness) of polyaniline (PANI) on CuS nanosheets (CuS@PANI) is obtained. When it was used as an anode for SIBs, the CuS@PANI delivered a high capacity of 500.0 mAh g^−1^ (closing to the theoretical capacity) and ultralong cycling life over 7500 cycles. Finite element analyses (FEA) reveal PANI layer with a high elongation (even up to 40%) [20] can buffer the volume expansion and suppress the surface pulverization of CuS. Besides, the positive NH^+^- groups in PANI can effectively bind the negative polysulfide, as evidenced by DFT calculation. Moreover, the PANI swollen by electrolytes can stabilize the SEI film, facilitate Na-ion transport, and confine the space for Cu particle growth during the conversion reaction. Such a universal strategy of using multi-functional coating can be extended to other MSs with improved performance for new-generation batteries.

## Experimental Section

### Synthesis of Erythrocyte-like CuS Microspheres

Copper (II) nitrate hydrate (Cu(NO_3_)_2_·3H_2_O) (0.5798) and thiourea (CH_4_N_2_S) (0.7308 g) were dissolved in 40 mL ethylene glycol. Next, the above solution was transferred into a 50 mL stainless steel autoclave and kept at 140 °C for 10 h. After natural cooling, the black products were filtered, rinsed with water and ethanol, and vacuum dried at 50 °C for 10 h.

### Synthesis of CuS@PANI Microsphere

0.1 g of the synthesized erythrocyte-like CuS microspheres, 0.005 g paratoluenesulfonic acid sodium salt (pTSA), and 20 μL aniline monomers were added to 40 mL deionized (DI) water under magnetic stirring for 2 h. Then, a solution dissolved by 0.114 g ammonium persulfate was dropped into the above-mixed solution under magnetic stirring for 3 h. All of the above experimental procedures were carried out in an ice bath. After the reaction, the products were collected by washing and drying at 50 °C for 10 h.

### Characterization

The crystal structure, surface feature, and morphology microstructure of the products were characterized using x-ray diffraction (XRD, Bruker AXS D8 Advance), scanning electron microscope (SEM, JSM-7001F, 10 kV), Fourier transform infrared spectra (FT-IR, Nicolet 380), transmission electron microscopy (TEM, JEM-2100, 200 kV), and x-ray photoelectron spectroscopy (XPS, ESCALAB 250 Xi).

### Electrochemical Testing

Electrochemical measurements were carried out in CR2032 coin cells, in which active materials (~ 2 mg, 80%), conductive carbon black (Super P, 10 wt%), and binder (polyvinylidene difluoride binder, PVDF, 10 wt%) were mixed and coated on a copper foil as the working electrode. A porous glass fiber film, 1 M sodium trifluomethanesulfonate (NaSO_3_CF_3_) in diethylene glycol dimethyl ether (DEGDME), and a sodium foil electrode were used as the separator, electrolyte, and counter/reference electrodes, respectively. The cyclic voltammetry (CV) curves between 0.01 and 3 V (vs. Na^+^/Na) and electrochemical impedance spectroscopy (EIS) were performed using an electrochemical workstation (Gamry Reference 3000). Galvanostatic charge/discharge tests were conducted on a Neware battery testing system (Neware, Shenzhen, China). *In situ* XRD measurements were performed in an electrochemical cell with a beryllium window, and the active material slurry was cast on an Al foil (See the schematic diagram of the cell device in Fig. S1), and the active material slurry was cast on an Al foil. For *ex situ* tests, the disassembled electrodes were washed in DEGDME, dried in Ar, transferred and tested with minimized exposure time in the air.

## Results and Discussion

**Fig. 1:**
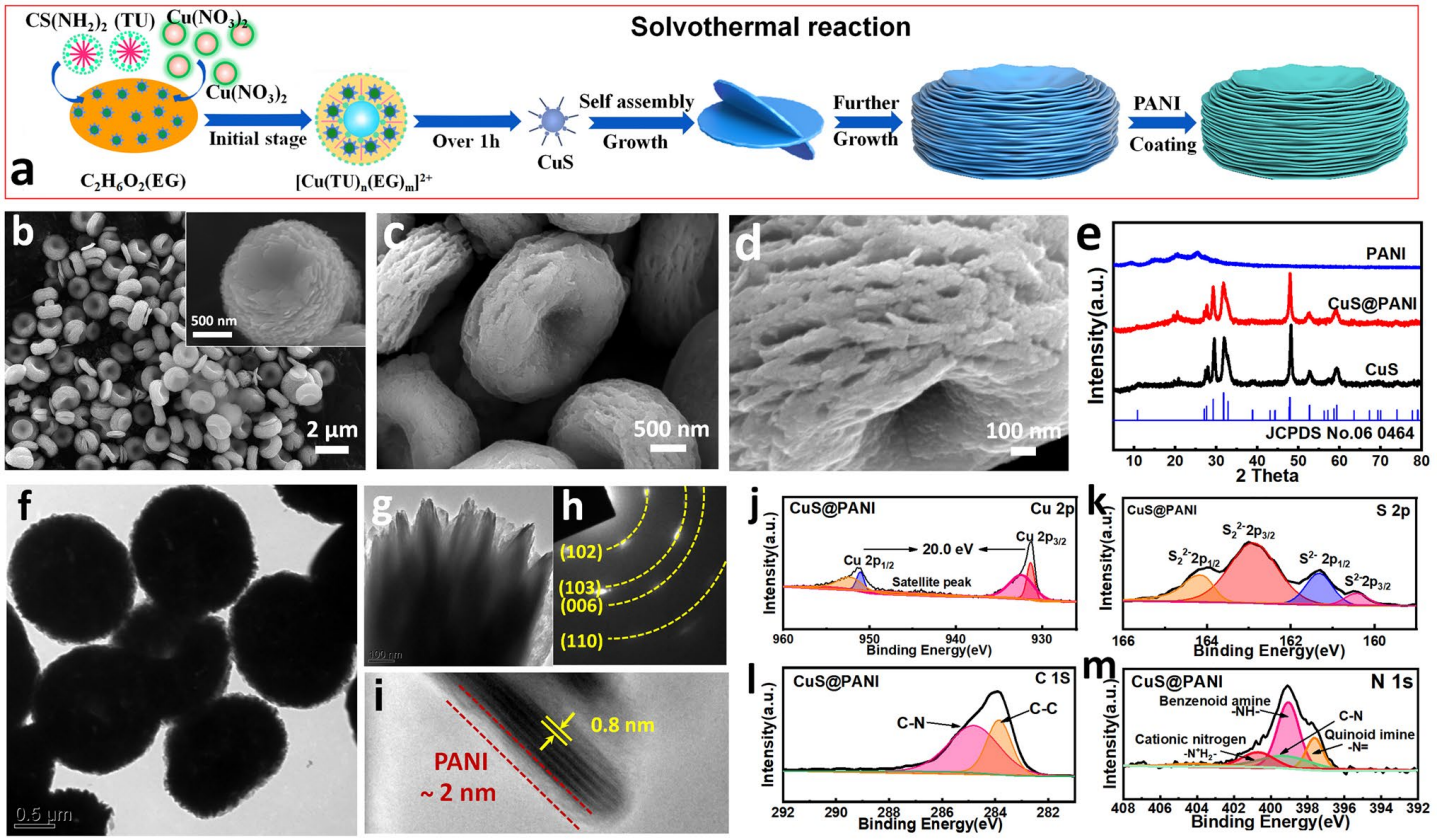
**a** Schematic illustration of the self-assembly growth of the erythrocyte-like CuS microsphere and the subsequent coating by PANI. The SEM images of the as-synthesized **b** CuS and **c**,** d** CuS@PANI. **e** XRD patterns of CuS, PANI, and CuS@PANI. **f-i** TEM image, SAED pattern, and HRTEM image of CuS@PANI. XPS spectra of CuS@PANI for **j** Cu 2*p*, **k** S 2*p*, ***l*** C 1*s*, and **m** N 1*s*

Figure 1a illustrates the growth mechanism and preparation process of CuS@PANI. First, erythrocyte-like CuS microspheres can be obtained by a solvothermal reaction of hydrated copper nitrate and thiourea (Tu) in ethylene glycol (EG) solvent for 10 h. In the initial stage of reaction, the Tu would coordinate with Cu^2+^ to produce [Cu(Tu)_*n*_(EG)_*m*_]^2+^ complexes (Fig. S2), and then the complex would decompose to form nuclei in further solvothermal process for over one hour [21]. The following nuclei will occur preferentially at the surface of CuS plate along the lower surface energy [22], which finally generates the intersectional circle CuS nanoplate. Meanwhile, the intersectional two CuS nanoplates will likely grow in the same direction based on the same lattice fringes in a conjoined place [23]. The SEM images of CuS intermediates obtained in time-dependent experiments (Fig. S3) show a small number of thin nanodisks with a diameter of ~ 1.0 μm at the initial reaction stage (2–4 h). With increasing the reaction time to 6 h, these nanodisks stacked with each other around a common center, forming a pancake-like shape. As the reaction time was further extended to 10 h, many more nanosheets crowded around to self-assemble into the uniform erythrocyte-like CuS microspheres with a diameter up to ~ 2.0 μm, as shown in Fig. 1b. For a long reaction time (over 10 h), the obtained samples still do not produce a complete sphere and maintain the same erythrocyte-like structure (Fig. S3). In addition, the reaction temperature plays a key role in the formation of CuS microspheres and the thickness of nanodisks (Fig. S4). Next, the erythrocyte-like CuS microspheres coated with an ultrathin PANI layer (Fig. 1c-d) were prepared based on a typical *in situ* polymerization method in an ice-bath solution containing aniline monomers and an oxidizing agent. The high-magnification SEM image (Fig. 1d) exhibits a rougher surface than pristine CuS microspheres, indicating that the PANI film was successfully grown on the surface of CuS nanosheets.

The sharp XRD peaks of CuS and CuS@PANI are matched well with the hexagonal CuS (JCPDS No. 06–0464) (Fig. 1e). No diffraction peaks of PANI can be found in CuS@PANI, indicating the low crystallinity and content of PANI. TEM measurement was carried out to analyze the detailed structure of CuS@PANI. Figure 1f-g displays the front and side views of the CuS@PANI, which agree well with the SEM results. The selected area electron diffraction (SAED) pattern with the polycrystalline diffraction rings can be indexed to the (102), (103), (006), and (110) planes of CuS (Fig. 1h). The HRTEM image (Fig. 1i) of the CuS@PANI demonstrates that the thickness of the PANI coating layer on CuS is ~ 2 nm, and the lattice spacing of 0.8 nm corresponds to the (002) plane of CuS. XPS analyses are further performed to confirm the chemical states of the elements involved in CuS@PANI. The Cu 2*p* spectrum (Fig. 1j) presents the Cu 2*p*_3/2_ (931.3 eV) and Cu 2*p*_1/2_ (951.3 eV) with a spin energy separation of 20.0 eV and an apparent satellite peak at 943.8 eV, which confirms the presence of Cu^2+^ [24]. In Fig. 1k, there are two doublets peaks at (161.3 and 160.4 eV) and (164.2 and 162.9 eV) assigned to S^2−^ and (S_2_)^2−^, respectively [25]. Figure 1l shows the C 1*s* spectrum with three peaks at 284.4, 285.8, and 287.8 eV corresponding to C–C, C–N, and C=N, respectively [26]. The N 1*s* spectrum in Fig. 1m is deconvoluted into four peaks, where three peaks at 398.5, 399.6, and 400.5 eV can be indexed to quinonoid imine (=N–), benzenoid imine (–NH–), and protonated amine (=NH^+^–), respectively [27]. And the rest of –NH_2_^+^– at 443 eV reflects the intrachain doping with p-toluenesulfonic acid (pTSA) on PANI. These results, combined with the IR analysis (Fig. S5), confirm that the PANI coating on CuS is the high conductive emeraldine salt [28].

**Fig. 2:**
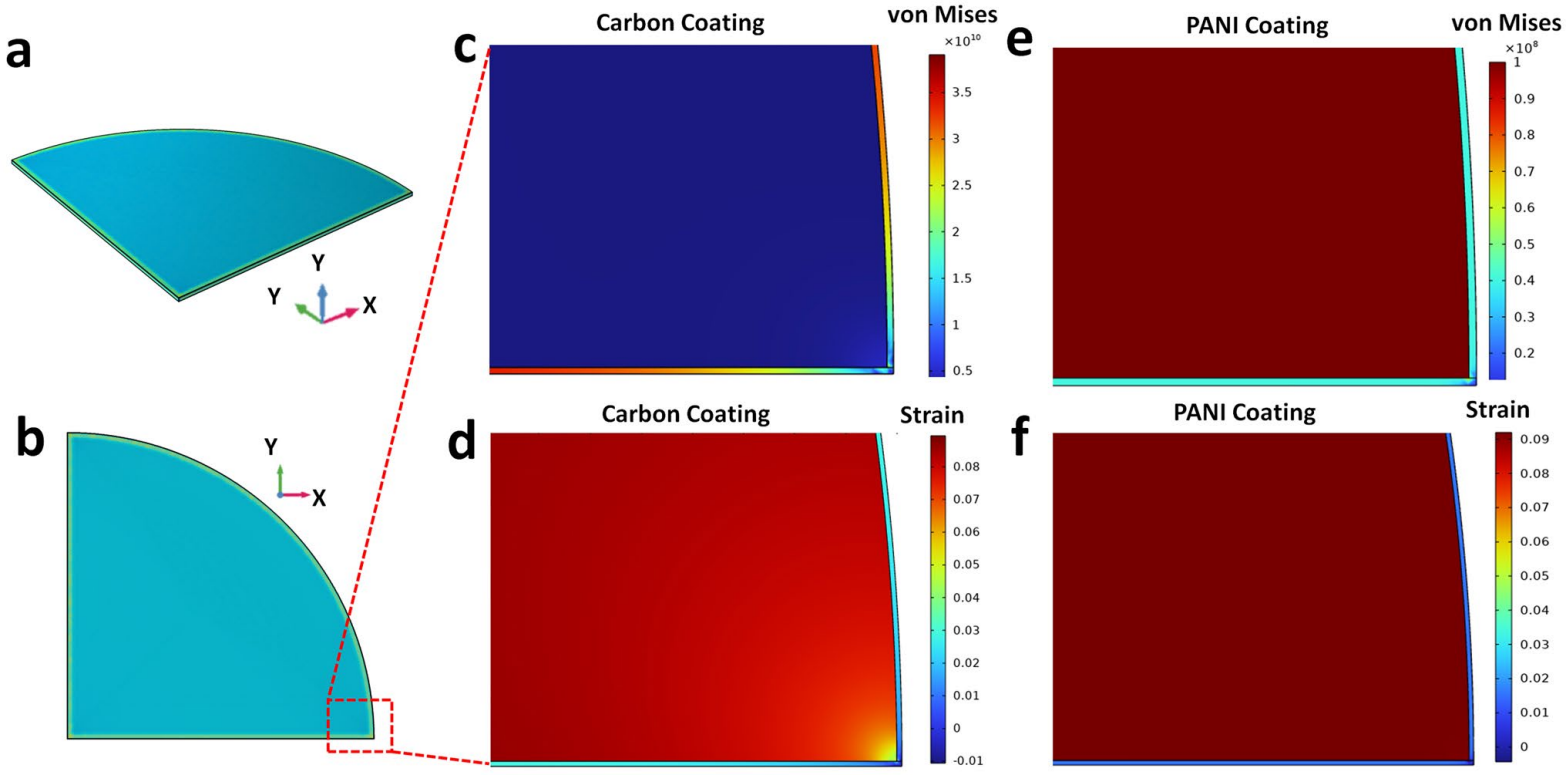
FEA simulations for comparing the strain and stress distributions of CuS@carbon and CuS@PANI coating

To fundamentally understand the advantage of elastic coating, we introduce finite element analyses (FEA) to compare the stress and strain distribution of CuS@PANI with CuS@Carbon film after the intercalation of Na ions. Given the lamellar structure unit of CuS microsphere, we built a similar fan-like CuS flake with a radius of 1 μm and a thickness of 100 nm (see the 3D and 2D views in Fig. 2a-b). The coating shell was set as 2 nm thickness, representing the amorphous carbon or PANI. The Young’s modulus and Poisson’s ratio of CuS were assumed as 60 GPa and 0.15, respectively, based on a survey of metal sulfides [29–32]. And those of the amorphous carbon film are 950 Gpa and 0.2 [33], respectively, as well as 0.9 Gpa and 0.3 for PANI [34–37]. The expansion coefficients of composites were appointed to remain a constant in the whole structure. During the volume expansion up to 10% caused by sodium-ion insertion, the carbon film reaches the highest stress of 39.1 GPa over its tensile strength (Fig. 2c) [38]. Under high stress, the carbon shell leads to a volume compression, inducing a severe compressive strain in sharp corners (hot spot), as shown in Fig. 2d. At the same volume expansion condition, the PANI shell obtains stress below 40 MPa (see the von Mises in Fig. 2e), smaller than its tensile stress of over 90 MPa [39–41]. The uniform train distributions (Fig. 2f) of CuS@PANI indicate CuS undergo a homogeneous expansion without strain concentration. Thus, the CuS@PANI has the potential to overcome the probable surface fracture and achieve excellent electrochemical and structural stability for SIBs.

**Fig. 3:**
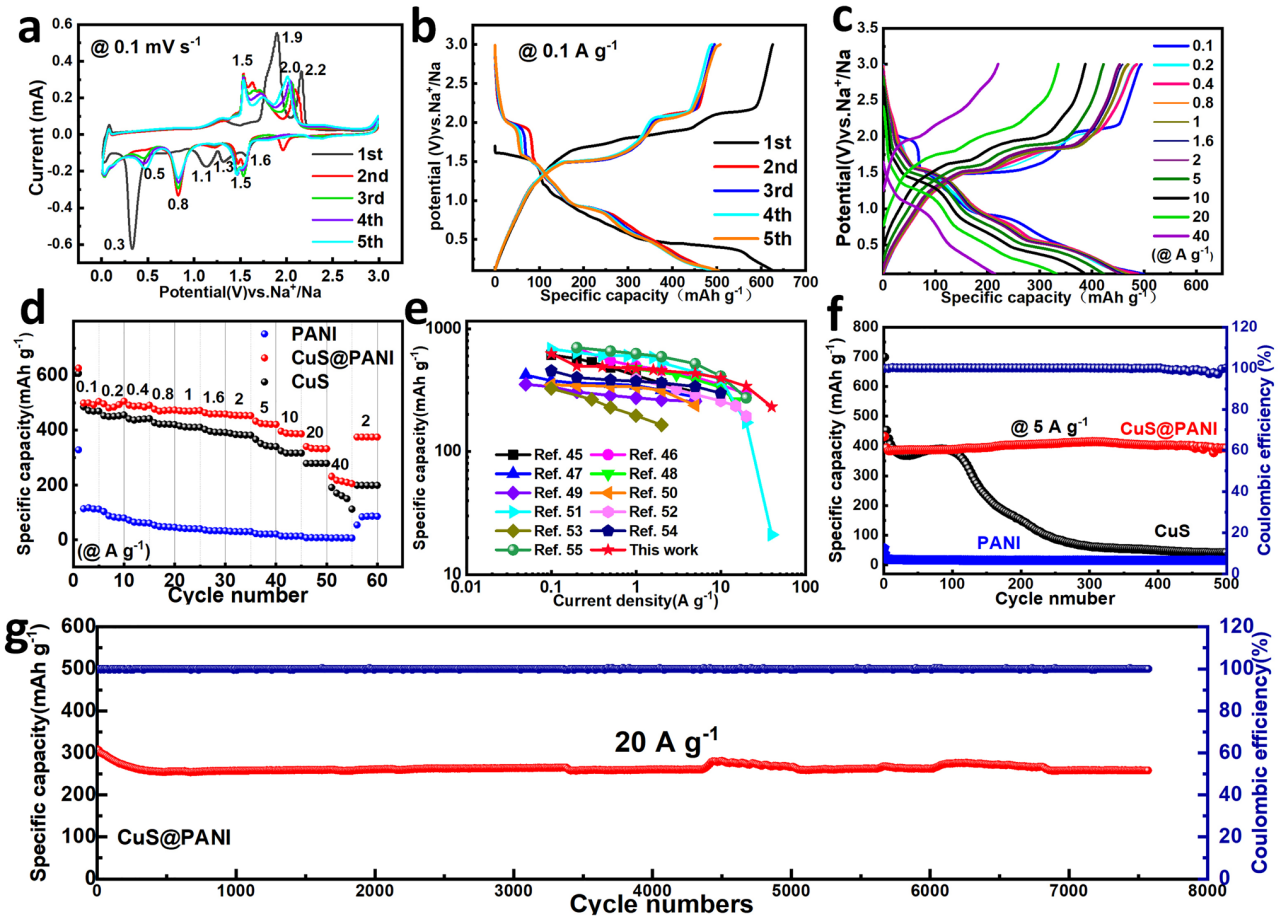
**a** First five CV curves at 0.1 mV s^−1^ and **b** GCD curves at 0.1 A g^−1^ for CuS@PANI electrode. **c** The GCD curves of CuS@PANI at different current densities and **d**,** e** the corresponding rate performance compared with CuS, PANI, and previously reported other CuS electrodes. **f** Cycling performance of CuS@PANI, CuS, and PANI at 5 A g^−1^, and **g** long cyclic stability of CuS@PANI at 20 A g^−1^

After optimization of the synthesis procedure by adjusting reaction time, temperature, and thickness of PANI film, the CuS@PANI samples prepared under a 10 h solvothermal process and the adding of 20 μL aniline monomers (Figs. S3, S4, and S6) were further systematically investigated as anode for SIBs. The initial five cyclic voltammograms (CV) curves of CuS@PANI are shown in Fig. 3a. In the first scan, the cathodic peaks of 1.6–1.1 V mainly correspond to the Na^+^ ion insertion into the CuS lattice to form Na_*x*_CuS (*x* < 0.5) [42]. And the strong irreversible peak at 0.3 V is associated with forming the solid electrolyte interphase (SEI) layer and the conversion of CuS to Cu and Na_2_S. The subsequent anodic peaks of 1.9 and 2.2 V are related to the multiple-phase transformations and desodiation process. In the following cycles, the cathodic peaks of 2.2–1.5 V correspond to the Na^+^ ion intercalation processes of Cu_2_S. The new reduction peaks of 0.8 and 0.5 V refer to the deeply sodiated Na_*x*_Cu_2_S (0.5 < *x* < 1) and conversion reaction with the generation of Cu and Na_2_S, respectively [43]. The 1.5–2.0 V oxidation peaks involve the multi-step desodiation process to produce the Cu_2_S [42, 44]. And the reduction/oxidation peaks are highly overlapped, demonstrating the reversible multi-step conversion reaction of Cu_2_S.

Figure 3b shows the first five galvanostatic charge/discharge (GCD) curves at 0.1 A g^−1^. The voltage plateaus of GCD curves agree well with the redox peaks of CV profiles. And the CuS@PANI electrode shows the first discharge capacity of 620.0 mAh g^−1^ with a coulombic efficiency value of nearly 100% and the reversible capacity of 500.0 mAh g^−1^ from the 2^nd^ to 5^th^ cycle with a slight capacity loss, which could be attributed to the irreversible formation of SEI and the phase transition from CuS to Cu_2_S. Pure CuS microspheres display similar CV and GCD curves to those of the CuS@PANI, suggesting the capacity contribution from the ultrathin coating layer of PANI can be ignored (Fig. S7). The erythrocyte-like CuS microspheres assembled by hierarchical nanosheets with highly conductive PANI coating will facilitate the transportation of electrolyte and electron conductivity, ensuring an excellent rate performance. As expected, the GCD curves of CuS@PANI even deliver stable charge/discharge plateaus at a ultrahigh current density of 40 A g^−1^ (Fig. 3c). The corresponding discharge capacities of 500.0, 484.1, 489.0, 471.5, 469.4, 459.4, 453.5, 422.7, 387.7, 332.3, and 214.5 mAh g^−1^ at 0.1- 40.0 A g^−1^ are obtained, respectively, which are superior to those of the individual CuS and PANI electrodes (Fig. 3d). In addition, the specific capacity of the CuS@PANI can quickly recover to 400 mAh g^−1^ as the current density is suddenly back to 2 A g^−1^, further demonstrating the high stability and reversibility of the CuS@PANI electrode. Figure 3e summarizes the rate performance of CuS-based anode materials reported recently in SIBs [43, 45–54]. It can be seen that CuS@PANI possesses a high specific capacity and a peerless advantage in ultrahigh rate performance. To evaluate the cycling stability of the CuS@PANI microspheres, we carried out the cycling test at a current density of 5 A g^−1^ within 0.01—3 V was carried out (Fig. 3f). The CuS@PANI cell retains a high reversible capacity of 393.3 mAh g^−1^ (over 99% of the 3^rd^ capacity) after 500 cycles with a high Coulombic efficiency of nearly 100%. While the bare CuS without PANI coating begins to decay seriously after 120 cycles, which can be attributed to the severe surface pulverization (see the *ex situ* SEM image in Fig. S8). The pure PANI electrode only displays a low specific capacity of ~ 17 mAh g^−1^. Additionally, the structural tolerance of CuS@PANI was further evaluated in a higher current density (20 A g^−1^), as shown in Fig. 3g. It is noteworthy that CuS@PANI still maintains 266 mAh g^−1^ with a high retention of 91% over 7500 cycles, superior to other metal sulfides electrodes in SIBs (Table S1), demonstrating the vital role of PANI in improving the cycling stability of CuS.

**Fig. 4:**
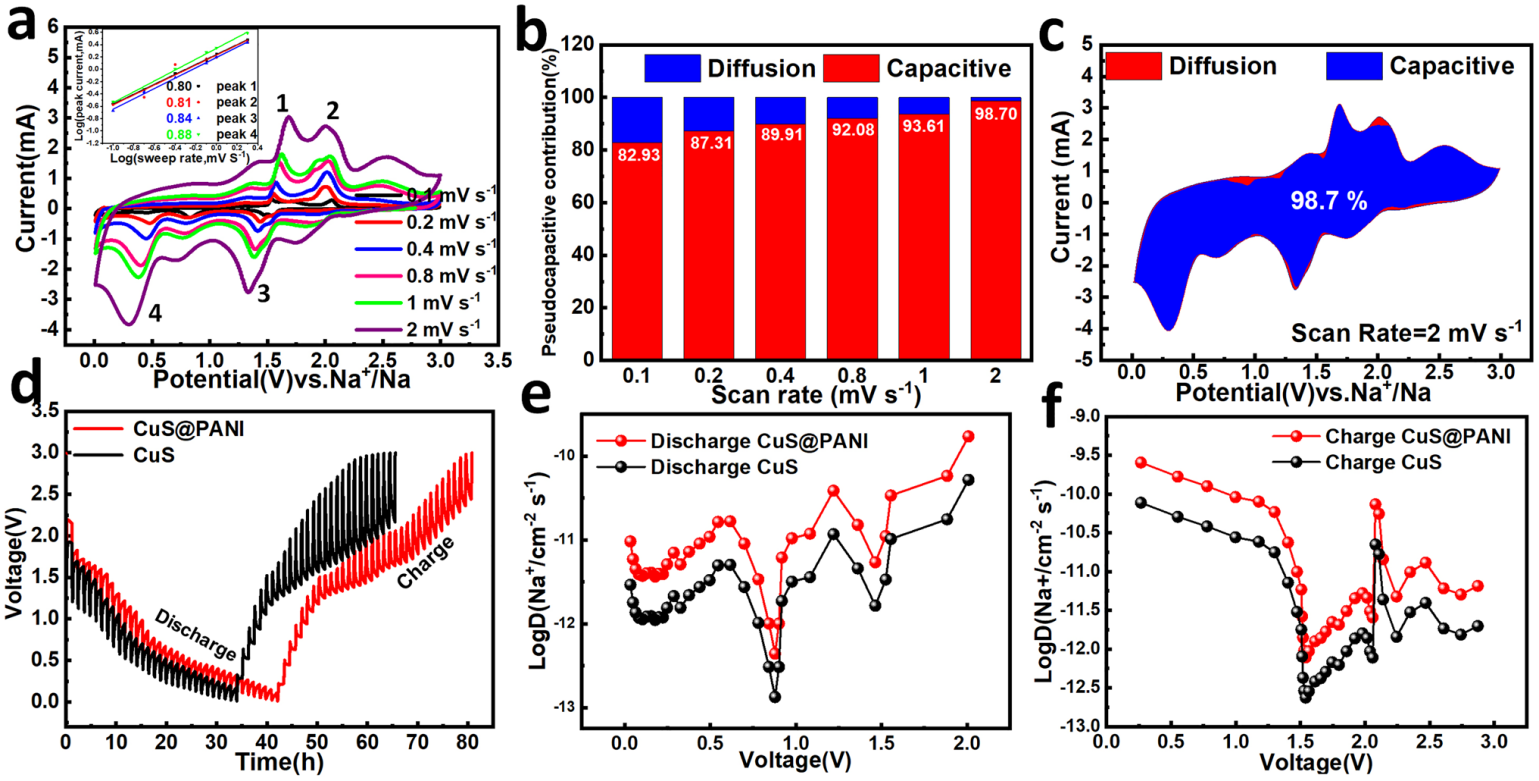
**a** CV curves at different scan rates, inset: its relationship of log(i) and log (*v*). **b** Capacitive and diffusion contribution calculated based on the CV results and **c** corresponding detailed shaded area of CV curve at 2 mV s^−1^. **d** GITT charge/discharge curves for CuS and CuS@PANI, and **e**,** f** the corresponding sodium-ion diffusion coefficient

To reveal the excellent rate performance of CuS@PANI, we used the CV test method to distinguish the typical capacitive contribution (Fig. 4a). According to the equation of log(*i*, peak currents) = blog(*v*, scan rates) + log(*a*) [50], the calculated *b* value are 0.80, 0.81, 0.84 and 0.88 for peaks 1 to 4, respectively (inset in Fig. 4a), which approach 1, demonstrating the capacitance dominant process. The equation of *i* = (*k*_1_*v,* capacitive part) + (*k*_2_*v*^0.5^, diffusion-controlled part) was employed to quantify its ratio of capacitive contribution. Figure 4b displays the increase in capacitive contribution with rising rates, e.g., as high as 98.7% of the pseudocapacitive contribution (blue area) at 2.0 mV s^−1^ (Fig. 4c), which exceeds the bare CuS microspheres (Fig. S9). Figure 4d shows the galvanostatic intermittent titration technique (GITT) measurement curves. Compared with CuS@PANI, the pure CuS show a significant polarization voltage in the charging process related to the desodiation process of Na_2_S to produce Cu_2_S. The corresponding sodium-ion diffusion coefficients (see the calculated method in Supporting Information and Fig. S10) of CuS@PANI are 1.8 × 10^–10^–4.5 × 10^–13^ cm^2^ s^−1^ in discharging process and 2.5 × 10^–10^–7.8 × 10^–13^ cm^2^ s^−1^ in charging process, respectively, outperforming the pure CuS in charge/discharge process. These results are supported by the EIS tests (Fig. S11), manifesting that the PANI coating lowers the charge transfer resistance and promotes the charge/ion transport at the interface.

**Fig. 5:**
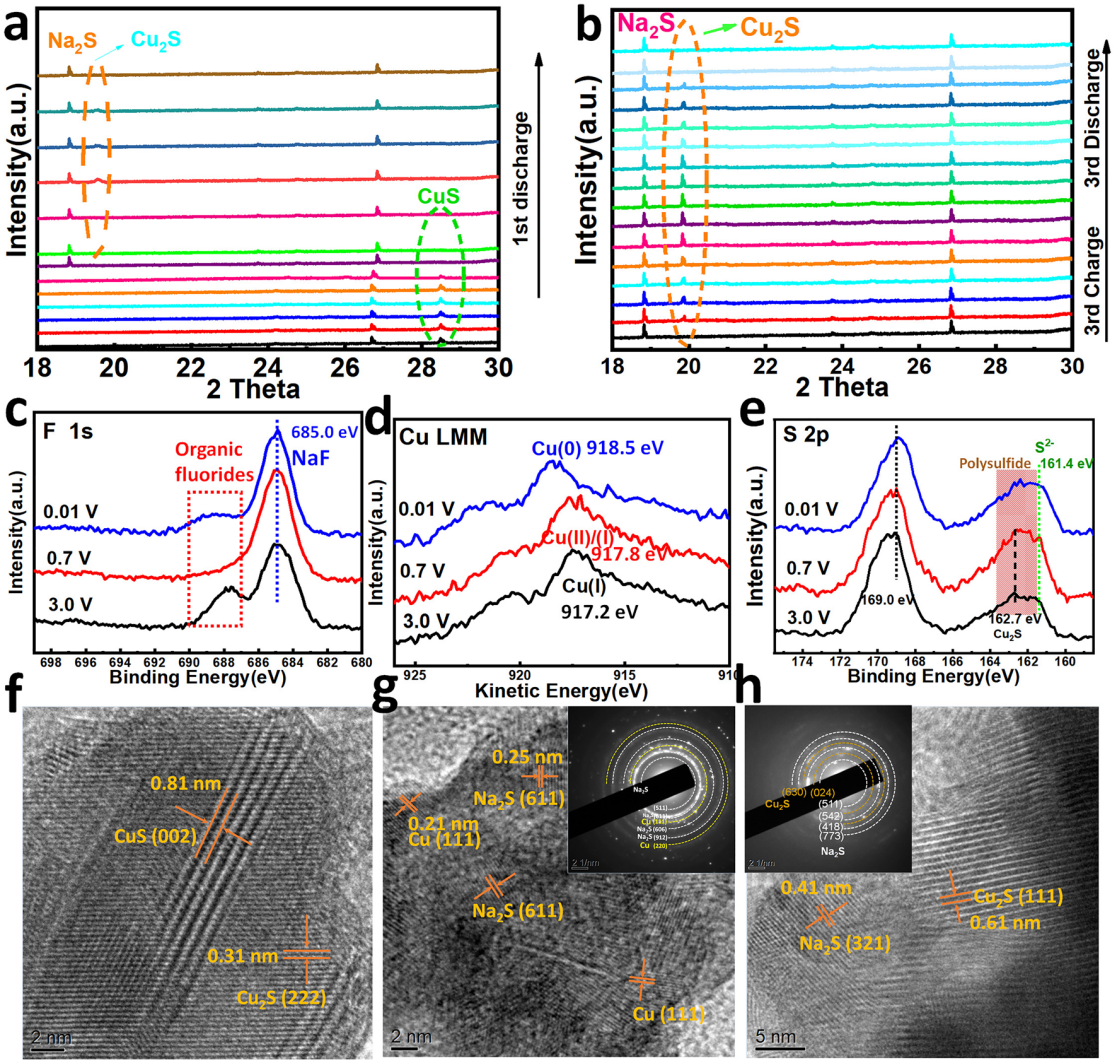
**a, b** *In situ* XRD patterns of CuS@PANI during the first discharge and the third CGD processes. The ex situ XPS analysis of CuS@PANI for **c** F 1* s*, **d** Cu LMM, **e** S 2*p* spectra at 0.01, 0.7 and 3.0 V states. The HRTEM images and SAED patterns of CuS@PANI electrode at different CGD states: **f** (0.7 V), **g** (0.01 V), and **h** (3.0 V)

To better understand the sodium-ion storage mechanism, in situ XRD, ex situ XPS, and TEM measurements were conducted to investigate the structural evolution of CuS@PANI. During the initial sodiation, the diffraction peak of CuS gradually decreases until it disappears (Fig. 5a), corresponding to the Na intercalation process (CuS + *x*Na^+^  + *x*e^−^ → Na_*x*_CuS). Then, the emergence of the weak peaks of Cu_2_S and Na_2_S can be attributed to a disproportionation reaction of Na_*x*_CuS → (*x*/2)Na_2_S + (*x*/2)Cu_2_S + (2 – *x*)CuS. In the following cycles (Fig. 5b), the Cu_2_S peaks appear and disappear periodically along with the GCD process, indicating a highly reversible conversion reaction of Cu_2_S + 2Na^+^  + 2e^−^ ↔ Na_2_S + 2Cu. The ever-present Na_2_S peak mainly stems from the overweight Na_2_S produced in the irreversible phase transformation [55]. The F 1*s* spectra at the different discharged and charged states (Fig. 5c) exhibit the bands at around 685.0 and 688.4 eV, corresponding to the formation of NaF and organic fluorides (SEI film), respectively [56]. That demonstrated the SEI layer begins to emerge as discharging from 0.7 to 0.01 V and get stronger in the subsequent charging to 3.0 V. As shown in Fig. 5d, the Cu LMM spectrum measured at the discharged 0.7 V state presents a broad peak at 917.8 eV near Cu(I), which indicates a fast phase transformation from Cu(II) to Cu(I) happened at 0.7 V. For the sample fully discharged to 0.01 V, the emerging Cu LMM Auger peak at 918.5 eV refers to the Cu(0), confirming the metal Cu generated in the conversion reaction [57]. After a full charging to 3.0 V, the prominent peak at 917.2 eV corresponds to the Cu(I), confirming the formation of Cu_2_S from the electrochemical oxidation of Cu(0). In the S 2*p* spectra (Fig. 5e), the dominant peak at around 169.0 eV corresponds to sulfate (from the electrolyte and partially oxidized material by air) [58]. The peak at 161.4 eV appearing in all states is assigned to Na_2_S [59], consistent with the in situ XRD results. The peak at 162.7 eV can be indexed to sulfide of Cu_2_S [60, 61]. Besides, the shadow area between 161.4 and 163.9 eV for the electrode at the three states involves the central-S atoms in polysulfide chains, suggesting the existence of polysulfide that cannot be completely transformed in the GCD process [61–63]. Furthermore, the *ex situ* HRTEM image of the discharged electrode at 0.7 V (Fig. 5f) reveals the intermediate state of the emergence of Cu_2_S (222) accompanied by the disappearance of CuS (002), demonstrating the phase transformation from CuS to Cu_2_S. The inverse fast Fourier transform (IFFT) image and geometric phase analysis (GPA) (as shown in Fig. S12) reveal the formation of crystallographic defects of edge and screw dislocation after intercalating Na^+^ ion, which induces the uniform tensile stress and compressive stress in the electrode materials. Deeply discharged to 0.01 V, the HRTEM image (Fig. 5g) presents the fully transformed products of the Na_2_S and Cu particles, evidenced by their SAED pattern inserted in the image. After charging up to 3.0 V (Fig. 5h), the main products of Cu_2_S and ever-presented Na_2_S can be found in the HRTEM image and corresponding SAED pattern.

**Fig. 6:**
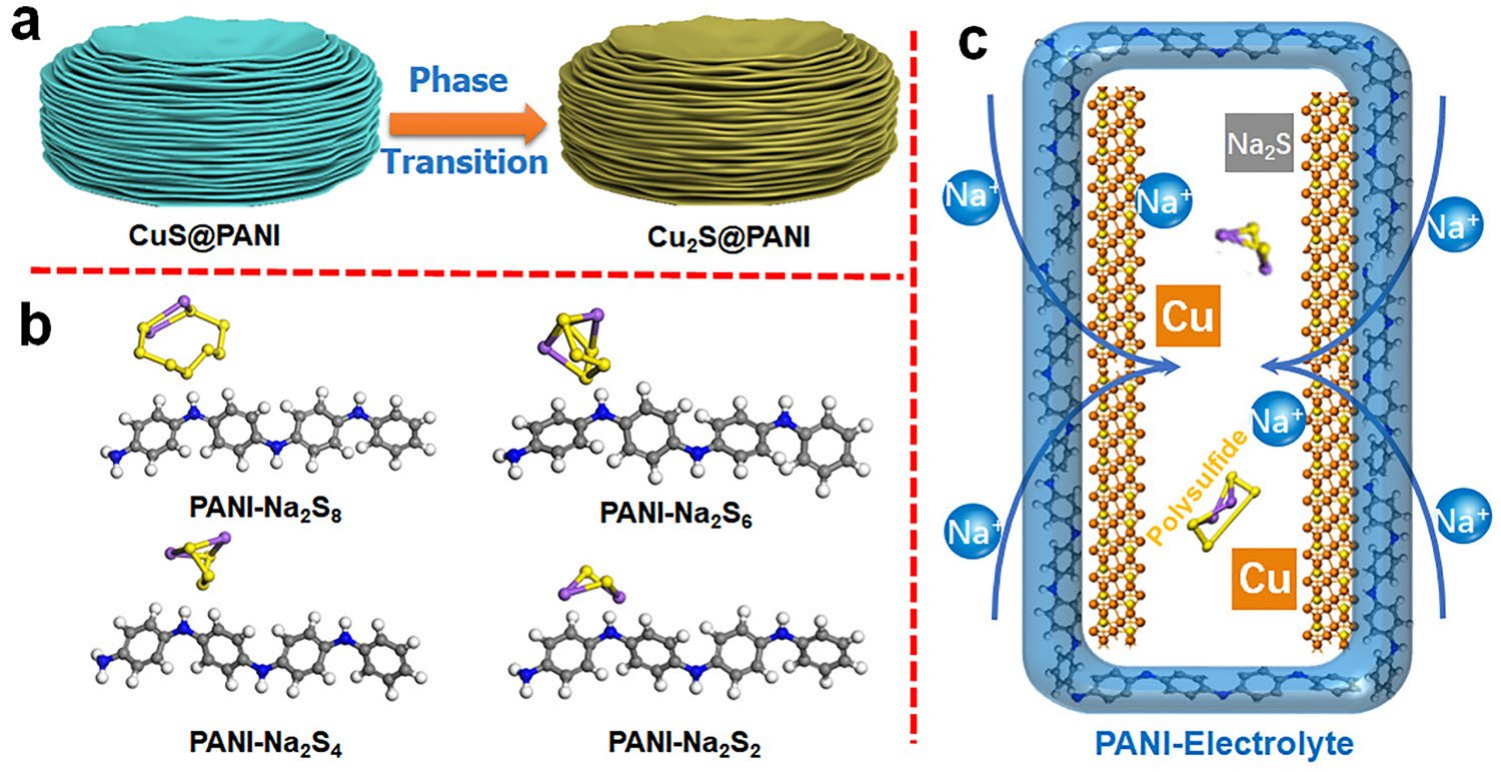
**a** Schematic illustration for phase transition from CuS@PANI to Cu_2_S@PANI, **b** adsorption configuration of polysulfide on PANI, and **c** the Cu_2_S encapsulated in PANI-electrolyte with improving Na-ion storage

Based on the above analysis, the mechanism of the excellent rate property and long cycling life of CuS@PANI is illustrated in Fig. 6. First, the CuS@PANI was transformed into Cu_2_S@PANI during the sodiation/desodiation process (Fig. 6a) and maintained its original morphology (Fig. S13). Meanwhile, the PANI rich in positive charge (=NH^+^–) can adsorb the sodium polysulfide, further evidenced by the density functional theory calculations, showing the adsorption energy (*E*_ad_) of Na_2_S_*x*_ (*x* = 2, 4, 6, 8) is −4.1, −2.5, −1.7, and −0.6 eV, respectively (Fig. 6b, the detailed computational method was shown in Supporting Information). The ultrathin PANI coating was swollen by electrolytes (see the thickness of PANI film increased by 20.4% after swelling in the electrolyte; meanwhile, the swollen PANI can restore to its initial state after a drying process, indicating its high elasticity, as shown in Fig. S14). Then, the PANI matrix with Na^+^ doping and taking in the corrosive HF can prevent the continued buildup of poorly conducting fluoride [64], resulting in a stable SEI layer suitable for ion transport (Fig. 6c). And the encapsulated space by PANI can not only contribute to buffering the volume expansion but also can confine the excessive growth of nanoparticles in conversion reaction (see the ex situ HRTEM observation of Fig. 5g, showing the size of Cu particles no more than 6 nm), ensuing little change in morphology and suppressing the surface pulverization upon cycling. Overall, the introduction of multi-functional PANI coating ensures efficient utilization of CuS electrodes, affording an outstanding comprehensive performance of high specific capacity, high rate, and ultralong cycle stability.

Furthermore, we also evaluated the electrochemical performance of hybrid sodium-ion capacitors (SIC) by using CuS@PANI as the anode and commercial active carbon (AC) as the cathode electrode (see the details in Fig. S15). Benefiting from the high-rate pseudocapacitive process in CuS@PANI anode, the CuS@PANI//AC SIC delivers a high energy density of 50 Wh kg^−1^ at a power density of 140 W kg^−1^ and retains 23 Wh kg^−1^ at an ultrahigh power density of 6250 W kg^−1^. The excellent performance of CuS@PANI//AC SIC is superior to that of previously reported SIC, such as SMGA (sulfur-doped Ti_3_C_2_T_*x*_/RGO)//AC (41 Wh kg^−1^ at 197 W kg^−1^ and 25 Wh kg^−1^ at 2473 W kg^−1^) [65], Bi-stacked MXene//AC (40 Wh kg^−1^ at 38 W kg^−1^) [66], NiCo_2_O_4_//AC (23.5 Wh kg^−1^ at 36 W kg^−1^) [67], and Na-TNT (sodium titanate nanotubes)//AC (33.6 Wh kg^−1^ at 120 W kg^−1^) [68]. Such CuS@PANI//AC SIC sustains a retention of 80.1% after 1000 GCD cycles at a current density of 5 A g^−1^ with a high coulombic efficiency of above 99.6%.

## Conclusions

In this work, erythrocyte-like CuS microspheres were prepared based on self-assembly growth via a solvothermal method, followed by the PANI coating to fabricate CuS@PANI composite. When used as anodes for SIBs, the CuS@PANI delivers an excellent rate capability (500 mAh g^−1^ at 0.1 A g^−1^, and 214.5 mAh g^−1^ at 40 A g^−1^) and superior cyclability of over 7500 cycles at 20 A g^−1^. These outstanding electrochemical performances can be mainly attributed to the contribution of multi-functional PANI. PANI coating on CuS buffers the volume expansion, suppresses the surface pulverization, and binds the negative polysulfide. Moreover, PANI swollen by electrolytes can stabilize the SEI layer, facilitate Na-ion transport and charge transfer at the electrode interface, and restrain further growth of nanoparticles in conversion reaction. Consequently, the convenient strategy can be extended to other MSs with overall performance improvement in SIBs.

## Supplementary Information

Below is the link to the electronic supplementary material.

Supplementary file1 (PDF 2166 KB)

## References

[1] Ding Y, Cano ZP, Yu A, Lu J, Chen Z (2019). Automotive Li-ion batteries: current status and future perspectives. Electrochem. Energy Rev..

[2] Yang J, Ju Z, Jiang Y, Xing Z, Xi B (2018). Enhanced capacity and rate capability of nitrogen/oxygen dual-doped hard carbon in capacitive potassium-ion storage. Adv. Mater..

[3] Wei Q, Li Q, Jiang Y, Zhao Y, Tan S (2021). High-energy and high-power pseudocapacitor–battery hybrid sodium-ion capacitor with Na^+^ intercalation pseudocapacitance anode. Nano-Micro Lett..

[4] Wei Q, DeBlock RH, Butts DM, Choi C, Dunn B (2020). Pseudocapacitive vanadium-based materials toward high-rate sodium-ion storage. Energy Environ. Mater..

[5] Xie F, Xu Z, Guo Z, Titirici MM (2020). Hard carbons for sodium-ion batteries and beyond. Prog. Energy.

[6] Lan Y, Yao W, He X, Song T, Tang Y (2020). Mixed polyanionic compounds as positive electrodes for low-cost electrochemical energy storage. Angew. Chem. Int. Ed..

[7] Yang J, Xiao S, Cui X, Dai W, Lian X (2020). Inorganic-anion-modulated synthesis of 2D nonlayered aluminum-based metal-organic frameworks as carbon precursor for capacitive sodium ion storage. Energy Storage Mater..

[8] Yang J, Wang X, Dai W, Lian X, Cui X (2021). From micropores to ultra-micropores inside hard carbon: toward enhanced capacity in room-/low-temperature sodium-ion storage. Nano-Micro Lett..

[9] Xiao Y, Lee SH, Sun YK (2017). The application of metal sulfides in sodium ion batteries. Adv. Energy Mater..

[10] Xiao Y, Zhao X, Wang X, Su D, Bai S (2020). A nanosheet array of Cu_2_Se intercalation compound with expanded interlayer space for sodium ion storage. Adv. Energy Mater..

[11] Li Z, Zhang Y, Li X, Gu F, Zhang L (2021). Reacquainting the electrochemical conversion mechanism of FeS_2_ sodium-ion batteries by operando magnetometry. J. Am. Chem. Soc..

[12] Geng H, Peng Y, Qu L, Zhang H, Wu M (2020). Structure design and composition engineering of carbon-based nanomaterials for lithium energy storage. Adv. Energy Mater..

[13] Yu XY, Yu L, Lou XW (2016). Metal sulfide hollow nanostructures for electrochemical energy storage. Adv. Energy Mater..

[14] Xiao Y, Xu Z, Liu Y, Peng L, Xi J (2017). Sheet collapsing approach for rubber-like graphene papers. ACS Nano.

[15] Güryel S, Hajgató B, Dauphin Y, Blairon JM, Miltner HE (2013). Effect of structural defects and chemical functionalisation on the intrinsic mechanical properties of graphene. Phys. Chem. Chem. Phys..

[16] Zhang Z, Zhang X, Wang Y, Wang Y, Zhang Y (2019). Crack propagation and fracture toughness of graphene probed by Raman spectroscopy. ACS Nano.

[17] Li P, Sun K, Ouyang J (2015). Stretchable and conductive polymer films prepared by solution blending. ACS Appl. Mater. Interfaces.

[18] Kalimuldina G, Nurpeissova A, Adylkhanova A, Adair D, Taniguchi I (2020). Morphology and dimension variations of copper sulfide for high-performance electrode in rechargeable batteries: a review. ACS Appl. Energy Mater..

[19] Yamakawa N, Jiang M, Grey CP (2009). Investigation of the conversion reaction mechanisms for binary copper(II) compounds by solid-state NMR spectroscopy and X-ray diffraction. Chem. Mater..

[20] Kitani A, Kaya M, Tsujioka SI, Sasaki K (1988). Flexible polyaniline. J. Polym. Sci. A Polym. Chem..

[21] Kumar P, Gusain M, Nagarajan R (2011). Synthesis of Cu_1.8_S and CuS from copper-thiourea containing precursors; anionic (Cl−, NO3−, SO42−) influence on the product stoichiometry. Inorg. Chem..

[22] Wu C, Yu SH, Antonietti M (2006). Complex concaved cuboctahedrons of copper sulfide crystals with highly geometrical symmetry created by a solution process. Chem. Mater..

[23] Wang S, Jiao S, Wang J, Chen HS, Tian D (2017). High-performance aluminum-ion battery with CuS@C microsphere composite cathode. ACS Nano.

[24] Ye M, Wen X, Zhang N, Guo W, Liu XY (2015). In situ growth of CuS and Cu_1.8_S nanosheet arrays as efficient counter electrodes for quantum dot-sensitized solar cells. J. Mater. Chem. A.

[25] An L, Zhou P, Yin J, Liu H, Chen F (2015). Phase transformation fabrication of a Cu_2_S nanoplate as an efficient catalyst for water oxidation with glycine. Inorg. Chem..

[26] Situ Y, Ji W, Liu C, Xu J, Huang H (2019). Synergistic effect of homogeneously dispersed PANI-TiN nanocomposites towards long-term anticorrosive performance of epoxy coatings. Prog. Org. Coat..

[27] Chen K, Zhang G, Xiao L, Li P, Li W (2021). Polyaniline encapsulated amorphous V_2_O_5_ nanowire-modified multi-functional separators for lithium–sulfur batteries. Small Methods.

[28] Dopico-García MS, Ares A, Lasagabáster-Latorre A, García X, Arboleda L (2014). Extruded polyaniline/EVA blends: enhancing electrical conductivity using gallate compatibilizers. Synth. Met..

[29] Kang J, Sahin H, Peeters FM (2015). Mechanical properties of monolayer sulphides: a comparative study between MoS_2_, HfS_2_ and TiS_3_. Phys. Chem. Chem. Phys..

[30] Rehman SU, Butt FK, Haq BU, AlFaify S, Khan WS (2018). Exploring novel phase of tin sulfide for photon/energy harvesting materials. Sol. Energy.

[31] Roldan A, Santos-Carballal D, Leeuw NH (2013). A comparative DFT study of the mechanical and electronic properties of greigite Fe_3_S_4_ and magnetite Fe_3_O_4_. J. Chem. Phys..

[32] Garcia-Mendez R, Smith JG, Neuefeind JC, Siegel DJ, Sakamoto J (2020). Correlating macro and atomic structure with elastic properties and ionic transport of glassy Li_2_S-P_2_S_5_ (LPS) solid electrolyte for solid-state Li metal batteries. Adv. Energy Mater..

[33] Yu MF, Lourie O, Dyer MJ, Moloni K, Kelly TF (2000). Strength and breaking mechanism of multiwalled carbon nanotubes under tensile load. Science.

[34] Sansiñena JM, Gao J, Wang HL (2003). High-performance, monolithic polyaniline electrochemical actuators. Adv. Funct. Mater..

[35] Makradi A, Ahzi S, Gregory RV (2000). Modeling of the mechanical response and evolution of optical anisotropy in deformed polyaniline. Polym. Eng. Sci..

[36] Pereira JN, Vieira P, Ferreira A, Paleo AJ, Rocha JG (2012). Piezoresistive effect in spin-coated polyaniline thin films. J. Polym. Res..

[37] Valentová H, Stejskal J (2010). Mechanical properties of polyaniline. Synth. Met..

[38] Li W, Cao K, Wang H, Liu J, Zhou L (2016). Carbon coating may expedite the fracture of carbon-coated silicon core–shell nanoparticles during lithiation. Nanoscale.

[39] Li ZF, Kang ET, Neoh KG, Tan KL (1997). Effect of thermal processing conditions on the intrinsic oxidation states and mechanical properties of polyaniline films. Synth. Met..

[40] Xiao HM, Zhang WD, Lv C, Fu SY, Wan MX (2010). Large enhancement in conductivity of polyaniline films by cold stretching. Macromol. Chem. Phys..

[41] Tan W, Stallard JC, Jo C, Volder MFLD, Fleck NA (2021). The mechanical and electrochemical properties of polyaniline-coated carbon nanotube mat. J. Energy Storage.

[42] Li H, Wang Y, Jiang J, Zhang Y, Peng Y (2017). CuS microspheres as high-performance anode material for Na-ion batteries. Electrochim. Acta.

[43] Liu R, Zhang Y, Wang D, Xu L, Luo S (2021). Microwave-assisted synthesis of self-assembled camellia-like CuS superstructure of ultra-thin nanosheets and exploration of its sodium ion storage properties. J. Electroanal. Chem..

[44] Hu Z, Liu Q, Chou S, Dou S (2017). Advances and challenges in metal sulfides/selenides for next-generation rechargeable sodium-ion batteries. Adv. Mater..

[45] Fang Y, Guan BY, Luan D, Lou XWD (2019). Synthesis of CuS@CoS_2_ double-shelled nanoboxes with enhanced sodium storage properties. Angew. Chem. Int. Ed..

[46] Zhao D, Yin M, Feng C, Zhan K, Jiao Q (2020). Rational design of N-doped CuS@C nanowires toward high-performance half/full sodium-ion batteries. ACS Sustain. Chem. Eng..

[47] Yang Z, Wu Z, Liu J, Liu Y, Gao S (2020). Platelet-like CuS impregnated with twin crystal structures for high performance sodium-ion storage. J. Mater. Chem. A.

[48] Zhang L, Hu Y, Liu Y, Bai J, Ruan H (2021). Tunable CuS nanocables with hierarchical nanosheet-assembly for ultrafast and long-cycle life sodium-ion storage. Ceram Int..

[49] Yang ZG, Wu ZG, Hua WB, Xiao Y, Wang GK (2020). Hydrangea-like CuS with irreversible amorphization transition for high-performance sodium-ion storage. Adv. Sci..

[50] Xiao Y, Su D, Wang X, Wu S, Zhou L (2018). CuS microspheres with tunable interlayer space and micropore as a high-rate and long-life anode for sodium-ion batteries. Adv. Energy Mater..

[51] Hu Y, Zhang L, Bai J, Liu F, Wang Z (2021). Boosting high-rate sodium storage of CuS via a hollow spherical nanostructure and surface pseudocapacitive behavior. ACS Appl. Energy Mater..

[52] An C, Ni Y, Wang Z, Li X, Liu X (2018). Facile fabrication of CuS microflower as a highly durable sodium-ion battery anode. Inorg. Chem. Front..

[53] Zhao W, Gao L, Yue L, Wang X, Liu Q (2021). Constructing a hollow microflower-like ZnS/CuS@C heterojunction as an effective ion-transport booster for an ultrastable and high-rate sodium storage anode. J. Mater. Chem. A.

[54] Zhao W, Wang X, Ma X, Yue L, Liu Q (2021). In situ tailoring bimetallic–organic framework-derived yolk–shell NiS_2_/CuS hollow microspheres: an extraordinary kinetically pseudocapacitive nanoreactor for an effective sodium-ion storage anode. J. Mater. Chem. A.

[55] Chung JS, Sohn HJ (2002). Electrochemical behaviors of CuS as a cathode material for lithium secondary batteries. J. Power Sources.

[56] Zhang Y, Zhu P, Huang L, Xie J, Zhang S (2015). Few-layered SnS_2_ on few-layered reduced graphene oxide as na-ion battery anode with ultralong cycle life and superior rate capability. Adv. Funct. Mater..

[57] Sung MM, Sung K, Kim CG, Lee SS, Kim Y (2000). Self-assembled monolayers of alkanethiols on oxidized copper surfaces. J. Phys. Chem. B.

[58] Liang X, Hart C, Pang Q, Garsuch A, Weiss T (2015). A highly efficient polysulfide mediator for lithium–sulfur batteries. Nat. Commun..

[59] C.D. Wagner, *Handbook of X-ray Photoelectron Spectroscopy*. (Perkin-Elmer Corporation, 1979).

[60] Deng S, Shen Y, Xie D, Lu Y, Yu X (2019). Directional construction of Cu_2_S branch arrays for advanced oxygen evolution reaction. J. Energy Chem..

[61] Fantauzzi M, Elsener B, Atzei D, Rigoldi A, Rossi A (2015). Exploiting XPS for the identification of sulfides and polysulfides. RSC Adv..

[62] Yu X, Manthiram A (2014). Room-temperature sodium–sulfur batteries with liquid-phase sodium polysulfide catholytes and binder-free multiwall carbon nanotube fabric electrodes. J. Phys. Chem. C.

[63] Hu M, Ju Z, Bai Z, Yu K, Fang Z (2020). Revealing the critical factor in metal sulfide anode performance in sodium-ion batteries: an investigation of polysulfide shuttling issues. Small Methods.

[64] Jin H, Xin S, Chuang C, Li W, Wang H (2020). Black phosphorus composites with engineered interfaces for high-rate high-capacity lithium storage. Science.

[65] Song F, Hu J, Li G, Wang J, Chen S (2021). Room-temperature assembled MXene-based aerogels for high mass-loading sodium-ion storage. Nano-Micro Lett..

[66] Kurra N, Alhabeb M, Maleski K, Wang CH, Alshareef HN (2018). Bistacked titanium carbide (MXene) anodes for hybrid sodium-ion capacitors. ACS Energy Lett..

[67] Ding R, Qi L, Wang H (2013). An investigation of spinel NiCo_2_O_4_ as anode for Na-ion capacitors. Electrochim. Acta.

[68] Yin J, Qi L, Wang H (2012). Sodium titanate nanotubes as negative electrode materials for sodium-ion capacitors. ACS Appl. Mater. Interfaces.

