# Supplementary material for: Elastic Buffering Layer on CuS Enabling High-Rate and Long-Life Sodium-Ion Storage
Source: Nanomicro Lett. 2022 Sep 23;14:193. doi: 10.1007/s40820-022-00924-3 (PMC9508307; doi:10.1007/s40820-022-00924-3)
Supplement: Supplementary file 1 — Supplementary file1 (PDF 2166 KB) [file 40820_2022_924_MOESM1_ESM.pdf]

Supporting Information for

Elastic Buffering Layer on CuS Enabling High-Rate and Long-Life Sodium-Ion Storage

Yuanhua Xiao[1], Feng Yue[1], Ziqing Wen[1], Ya Shen[1], Dangcheng Su[1], Huazhang Guo[2], Xianhong Rui[3], Liming Zhou[1, *], Shaoming Fang[1, *], and Yan Yu[4, *]

[1] Key Laboratory of Surface and Interface Science and Technology, Zhengzhou University of Light Industry, Zhengzhou 450002, P. R. China

[2] Institute of Nanochemistry and Nanobiology, School of Environmental and Chemical Engineering, Shanghai University, Shanghai 200444, P. R. China

[3] Institute School of Materials and Energy Guangdong University of Technology Guangzhou 510006, P. R. China

[4] Hefei National Research Center for Physical Sciences at the Microscale, Department of Materials Science and Engineering, National Synchrotron Radiation Laboratory, CAS Key Laboratory of Materials for Energy Conversion, University of Science and Technology of China. Hefei, Anhui 230026, P. R. China

*Corresponding authors. E-mail: zlming1212@126.com (Liming Zhou) mingfang@zzuli.edu.cn (Shaoming Fang); yanyumse@ustc.edu.cn (Yan Yu)

S1 GITT Measurement

During the GITT measurement, the cell was discharged or charged at 100 mA g^{-1} for 10 min and then rested for 60 min to reach the steady-state voltage (Es). The Na ion diffusivity D^{GITT} can be obtained via the formula [S1].

$$D = \frac{4}{\pi\tau}\left(\frac{m_A V_m}{M_A S}\right)^2 \left(\frac{\Delta E_s}{\Delta E_\tau}\right)^2 \left(\tau \ll \frac{L^2}{D}\right) \quad \text{(S1)}$$

where τ denotes the constant current pulse time, m_A, M_A, and V_m are the mass, molar weight, and molar volume of CuS@PANI, respectively, S is the area of the electrode-electrolyte interface, ΔEs is the change of Es obtained by subtracting the original voltage (E_0) from the steady-state voltage (Es), and ΔEτ is the total change of cell voltage during a constant current pulse τ excluding the IR-drop, and L (cm) is the diffusion length.

S2 Density Functional theory (DFT) Calculation Methods

The adsorption energies (E_{ads}) of Na_2S_x on PANI were calculated using the $DMol^3$ package[S2]. To fully optimize the finite molecular models, the standard Perdew-Burke-Ernzerhof (PBE) generalized gradient approximation (GGA)[S3] with Grimme's long-range dispersion correction was used for the exchange-correlation functional. All geometry optimizations and energy calculations were performed on a plane-wave basis sets with an energy cutoff of 400 eV. The double numeric plus polarization (DNP) basis sets were employed for all calculations. The convergence tolerance of the energy was set to 10^{-5} Hartree (1 Hartree = 27.21 eV), and the maximal allowed displacement and force were 0.005 Å and 0.002 hartree/Å, respectively. In addition, the models of PANI were composed of four monomers.

The E_{ads} values of Na_2S_x on PANI were calculated by:

$$E_{ads}=E_{sys}-E_{Na2Sx}-E_{PANI} \quad (S2)$$

E_{sys}, $E_{Na2Sx,}$ and E_{PANI} are the total energies of the adsorption systems, the adsorbate of Na_2S_x, and the PANI, respectively.

Table S1 A comparison of the electrochemical performance of our PANI@CuS with other metal sulfides anodes for SIBs

Materials	Rate capability	Cyclic stability	Refs.
This work	231.1493 mAh g^{-1} at 40 A g^{-1}	258.3444 mAhg^{-1} at 20 A g^{-1} (after 7576cycles)	This work
VS_4-rGO	192.2 mAh g^{-1} at 0.8 A g^{-1}	240.8 mAh g^{-1} at 0.1 A g^{-1} (after 50 cycles)	[S4]
SnS-C	145 mAh g^{-1} at 1 A g^{-1}	260 mAh g^{-1} at 0.1 A g^{-1} (after 300 cycles)	[S5]
SnS-MWCNT	410 mAh g^{-1} at 500 mA g^{-1}	391 mAh g^{-1} at 0.1 A g^{-1} (after 50 cycles)	[S6]
$CoMoS_3$	349 mAh g -1 at 10 A g -1	411 mAh g^{-1} at 2 A g^{-1} (after 300 cycles)	[S7]
FeS/N-C	260 mAh g^{-1} at 4 A g^{-1}	511 mAh g^{-1} at 0.2 A g^{-1} (after 100 cycles)	[S8]
CoS_2@N,Sdoped carbon	288 mAh g^{-1} at 2 A g^{-1}	510 mAh g^{-1} at 0.1 A g^{-1} (after 100 cycles)	[S9]
MoS_2 nanoflowers on N-doped graphene	201 mAh g^{-1} at 50 A g^{-1}	390 mAh g^{-1} at 1 A g^{-1} (after 1000 cycles)	[S10]
Hierarchical MoS_2 Hollow Architectures	226 mAh g^{-1} at 5 A g^{-1}	267 mA h g^{-1} at 1 A g^{-1} (after 125 cycles)	[S11]
MoS_2/C-MWCNT	324 mA h g^{-1} at 20 A g^{-1}	416 mA h g^{-1} at 2 A g^{-1} (after 1000 cycles)	[S12]
MoS_2/Graphene	201 mA h g^{-1} at 50 A g^{-1}	421 mA h g^{-1} at 0.3 A g^{-1} (after 250 cycles)	[S13]
MoS_2 Nanosheets /Nitrogen-Doped Graphene	141 mA h g^{-1} at 12.8 A g^{-1}	198 mAh g^{-1} at 1 A g^{-1} (after 1000 cycles)	[S14]
ANDC/MoS_2	409 mA h g^{-1} at 5 A g^{-1}	496 mAh g^{-1} at 1 A g^{-1} (after 1000 cycles)	[S15]
Nb_2CT_x@MoS_2@C	260 mA h g^{-1} at 40 A g^{-1}	403 mAh g^{-1} at 1 A g^{-1} (after 2000 cycles)	[S16]
MoS_2/SnS@C	325 mA h g^{-1} at 15 A g^{-1}	292 mAh g^{-1} at 5 A g^{-1} (after 2000 cycles)	[S17]
TiO-1T-MoS_2 NFs	288 mA h g^{-1} at 20 A g^{-1}	501 mAh g^{-1} at 1 A g^{-1} (after 700 cycles)	[S18]
P-MoS_2@C/CNTP	150 mA h g^{-1} at 5 A g^{-1}	249 mAh g^{-1} at 1 A g^{-1} (after 1200 cycles)	[S19]
(H-WS_2@NC	254 mA h g^{-1} at 8 A g^{-1}	375 mAh g^{-1} at 1 A g^{-1} (after 1000 cycles)	[S20]
NGQDs-WS_2/3DCF	211.4 mA h g^{-1} at 5 A g^{-1}	392.1 mAh g^{-1} at 0.2 A g^{-1} (after 1000 cycles)	[S21]
WS_2/CNT-rGO ordered 3D aerogel	47.2 mA h g^{-1} at 10 A g^{-1}	252.9 mAh g-1 at 0.2 A g^{-1} (after 100 cycles)	[S22]
WS_2 nanosheets/N-doped carbon	258 mA h g^{-1} at 1 A g^{-1}	~200 mAh g^{-1} at 0.1 A g^{-1} (after 100 cycles)	[S23]
WS_2@NC	151 mA h g^{-1} at 5 A g^{-1}	70 mAh g^{-1} at 5 Ag^{-1} (after 500 cycles)	[S24]
WS_2/C	81 mA h g^{-1} at 5 A g^{-1}	219 mAh g^{-1} at 0.5 A g^{-1} (after 300 cycles)	[S25]
WS_2@graphene	94 mAh g^{-1} at 0.64 A g^{-1}	94 mAh g^{-1} at 0.64 A g^{-1} (after 500 cycles)	[S26]

WS_2-3D RGO	287 mAh g^{-1} at 0.9 A g^{-1}	334 mAh g^{-1} at 0.2 A g^{-1} (after 200 cycles)	[S27]
SnS_2 NP/TiO_2@C	305.2 mAh g^{-1} at 5 A g^{-1}	338.2 mAh g^{-1} at 5 A g^{-1} (after 600 cycles)	[S28]
SnS_2/C Nanospheres	360h mAh g^{-1} at 1A g^{-1}	600 mAh g^{-1} at 0.05 A g^{-1} (after 100 cycles)	[S29]
2D SnS_2 nanosheets	435 mAh g^{-1} at 2 A g^{-1}	647 mAh g^{-1} at 0.1 A g^{-1} (after 50 cycles)	[S30]
SnS_2/rGO	337 mAh g^{-1} at 12.8 A g^{-1}	400 mAh g^{-1} at 0.8 A g^{-1} (after 1000 cycles)	[S31]

S3 Supplementary Figures

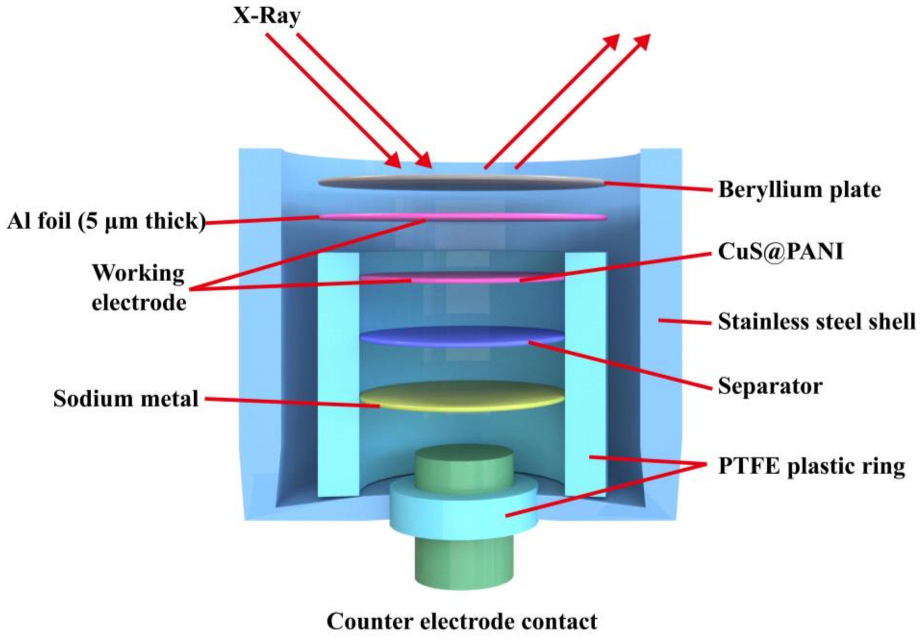

Fig. S1 Schematic illustration of the in-situ XRD cell structure

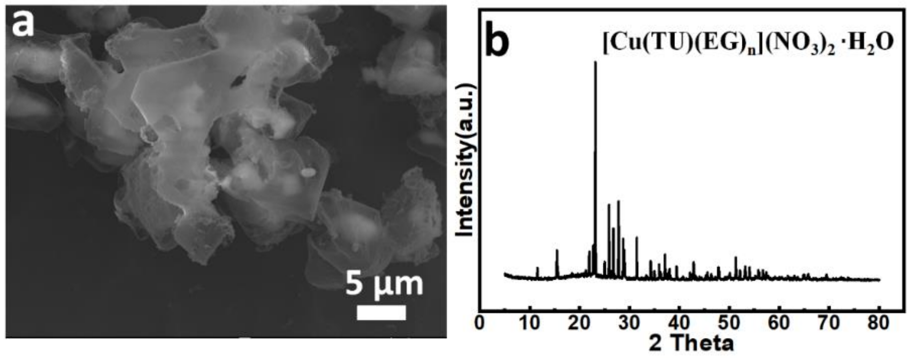

Fig. S2 The SEM image and XRD pattern of obtained samples in 40 min

In the initial stage of reaction, the Tu would coordinate with Cu^{2+} to produce $[Cu(\mathit{Tu})_n(\mathit{EG})_m]^{2+}$ complexes [S32], and then the complex would decompose to form nuclei in a further solvothermal process of over one hour. The chemical reactions can be expressed as follow:

$$Cu^{2+} + n\mathit{Tu} + m\mathit{EG} \rightarrow [Cu(\mathit{Tu})_n(\mathit{EG})_m]^{2+}$$

$$[Cu(\mathit{Tu})_n(\mathit{EG})_m]^{2+} \rightarrow CuS$$

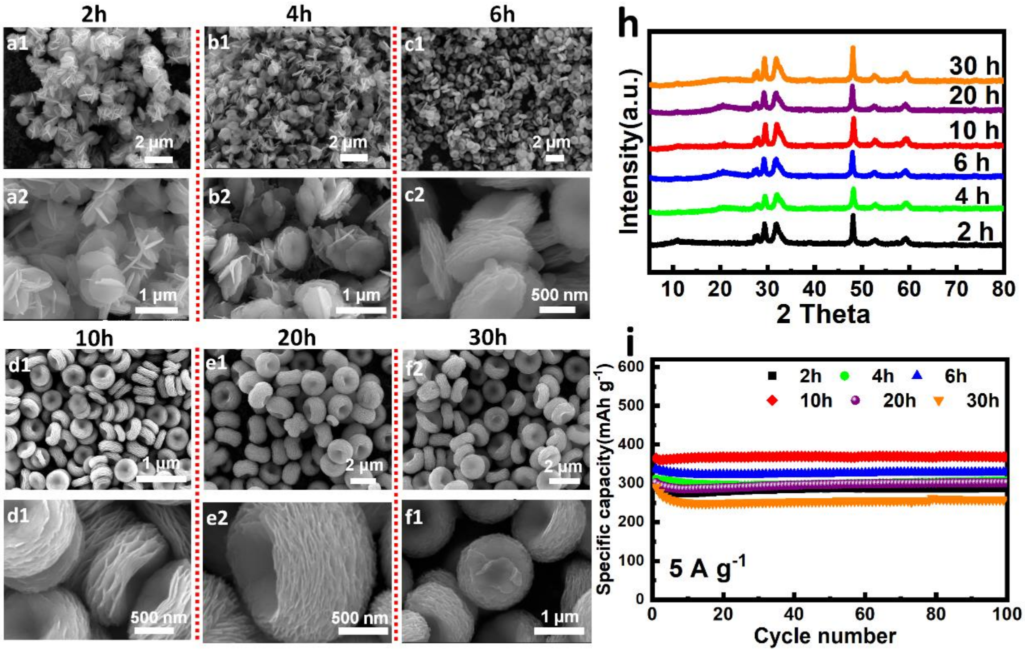

Fig. S3 (**a-f**) SEM images of CuS intermediates obtained by the different reaction time and (**h**) the corresponding XRD patterns and the evaluation of SIBs performance at 5 A g^{-1} (**i**)

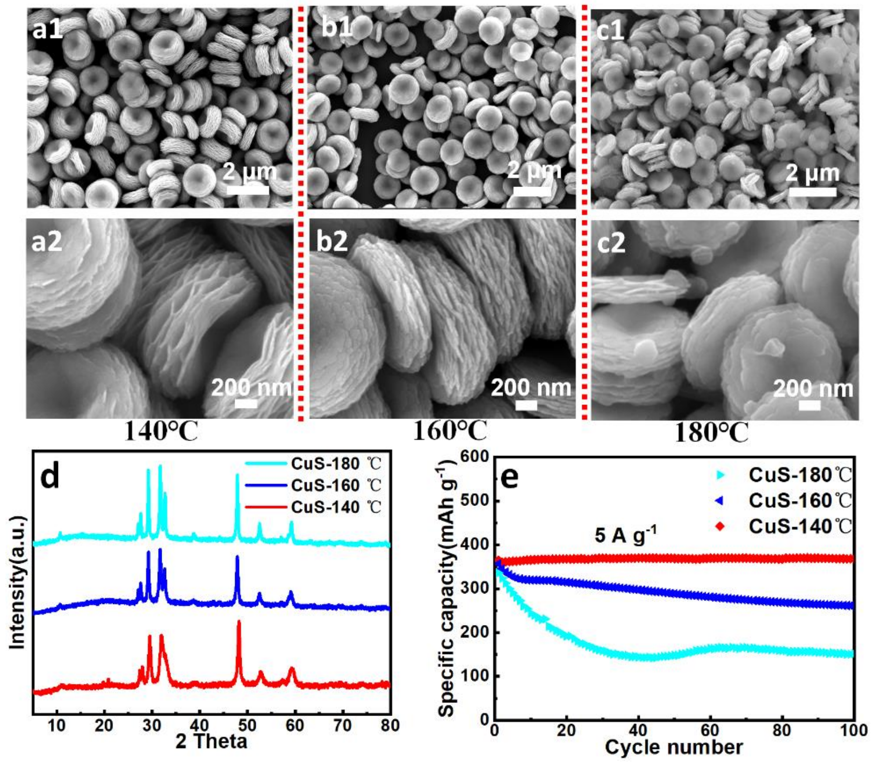

Fig. S4 (**a-c**) SEM images, XRD patterns (**d**), and cycling performance at 5 A g^{-1} of CuS samples obtained at different reaction temperatures (**e**)

It is worth noting that the CuS microspheres can not be obtained at a reaction temperature lower than 140 °C. And the product CuS-140 °C get a lower crystallinity of 72.9% at the 2θ of 32.7° (006) plane referring to layer thickness calculated via Jade 6.0 software (here CuS-160 °C and CuS-180 °C is 85.5% and 86.3%, respectively). The results indicate the nanosheets of CuS-140 °C get a thinner thickness, which agrees well with the SEM observation.

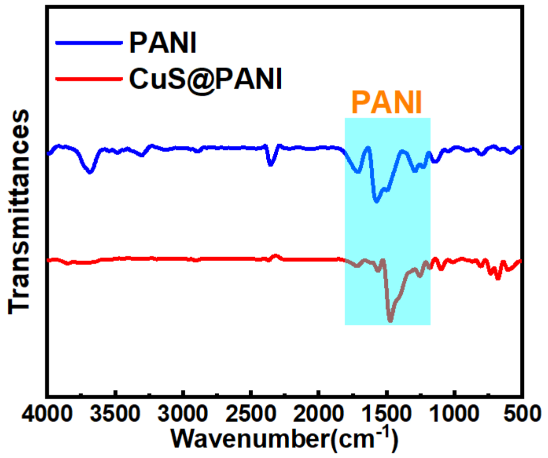

Fig. S5 FT-IR spectra of pure PANI and CuS@PANI

The pure PANI sample shows a series of characteristic peaks at 1135, 1294, 1491, and 1577 cm^{-1}. The absorption peaks at 1577 and 1491 cm^{-1} were associated with the C=C stretching vibrations in the quinonoid and benzenoid rings. The peaks at 1294 and 1135 cm^{-1} were attributed to C-N stretching of secondary amines and N=Q=N (Q: quinonoid structure) respectively. The characteristic peaks above are in agreement with the previous reports of PANI. The obvious redshift in the characteristic bands of PANI@CuS can be attributed to the interaction between PANI and CuS, which leads to weaken N-H, C-N, and N-Q-N bonds [S33].

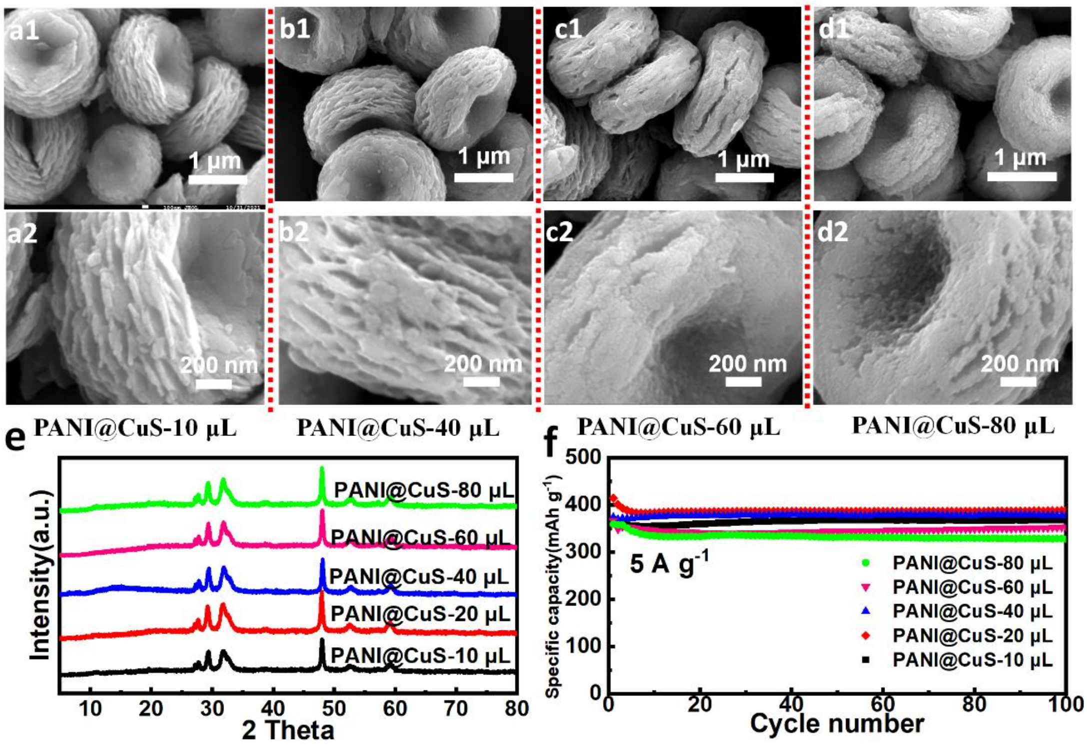

Fig. S6 SEM images of CuS@PANI microspheres with different amounts of ANI: (**a**) 10 μL, (**b**) 40 μL, (**c**) 60 μL, and (**d**) 80 μL. (**e**) The corresponding XRD patterns and the initial 100 cycling performance at 5 A g^{-1}

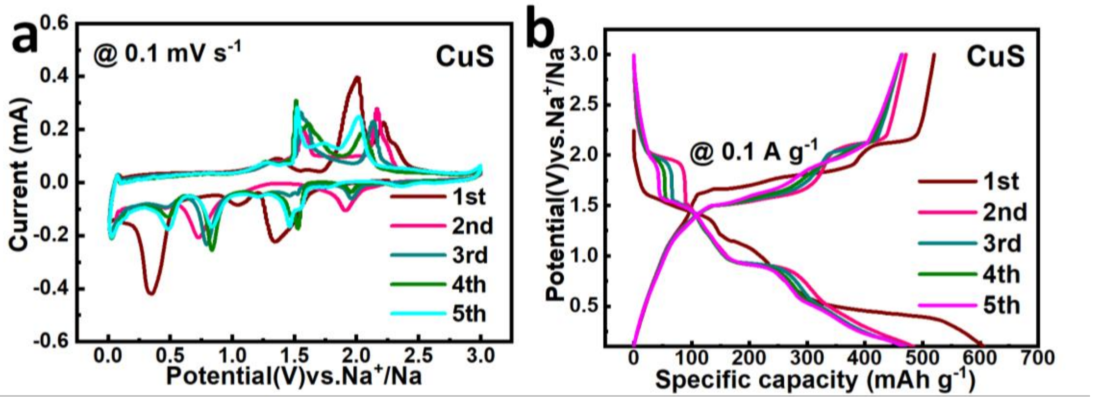

Fig. S7 (**a**) CV and (**b**) GCD curves of pure CuS microspheres during the first five cycles

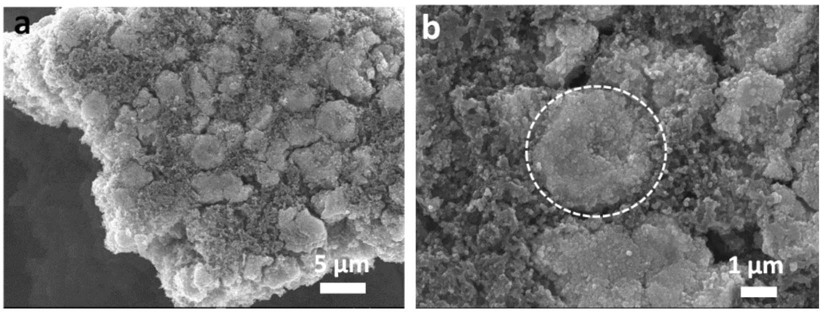

Fig. S8 Ex-situ images of the bare CuS microspheres after decay

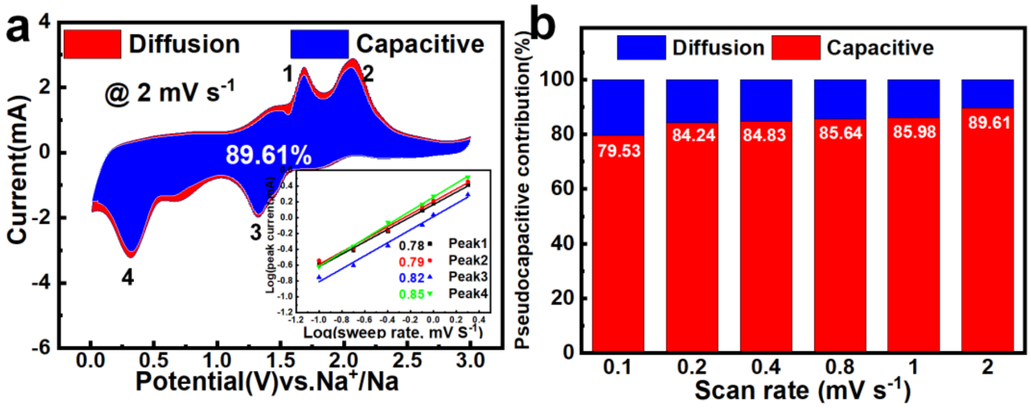

Fig. S9 Capacitive, diffusion contribution and corresponding *b* value calculated based on the CV curves for pure CuS microsphere

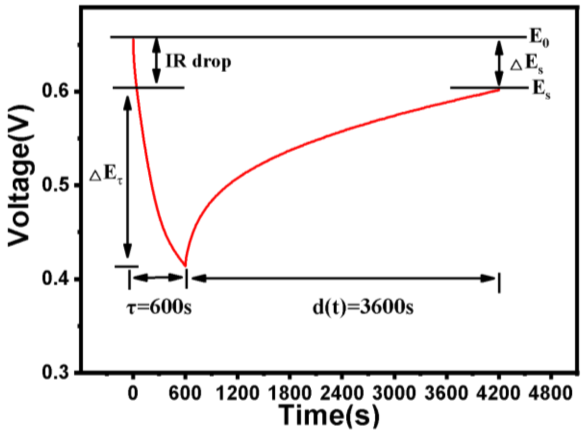

Fig. S10 An individual GITT curve of the CuS@PANI during the discharge process

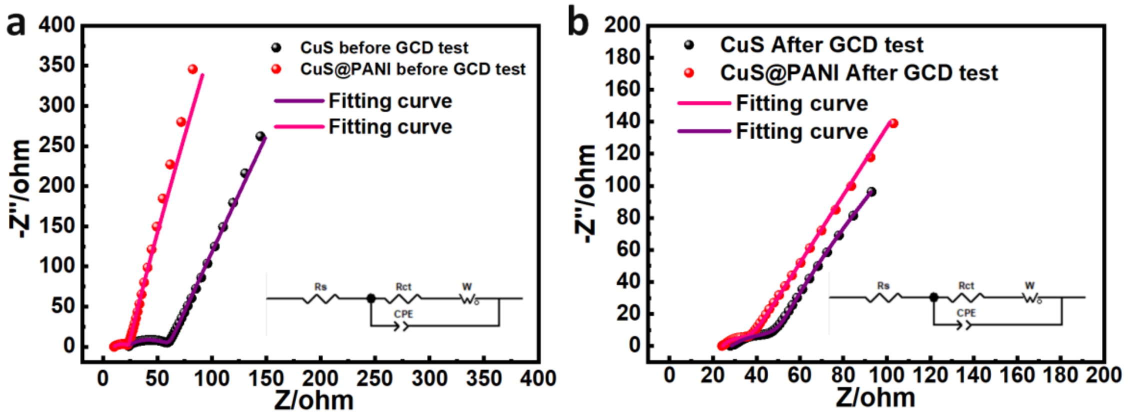

Fig. S11 Electrochemical impedance spectra for CuS@PANI and CuS (**a**) before the galvanostatic charge/discharge (GCD) and (**b**) after the GCD tests. Frequency range: 0.01Hz to 100 kHz. The inset in (**a**, **b**) is the equivalent circuit used for fitting the EIS data. Rs: electrolyte resistance. Rct: charge transfer resistance. W: Warburg impedance. CPE: constant phase element

It can be seen that at the high frequencies range, the CuS@PANI with a smaller semicircle diameter before/after galvanostatic charge-discharge (GCD), indicating the CuS@PANI has a lower R_{ct} (32 and 13 Ω) than that of CuS electrode 38 and 24 Ω) before/after GCD. Meanwhile, the Cu_2S with a lower band gap of 1.21 eV than CuS (2.42 eV) has a higher conductivity [S34].

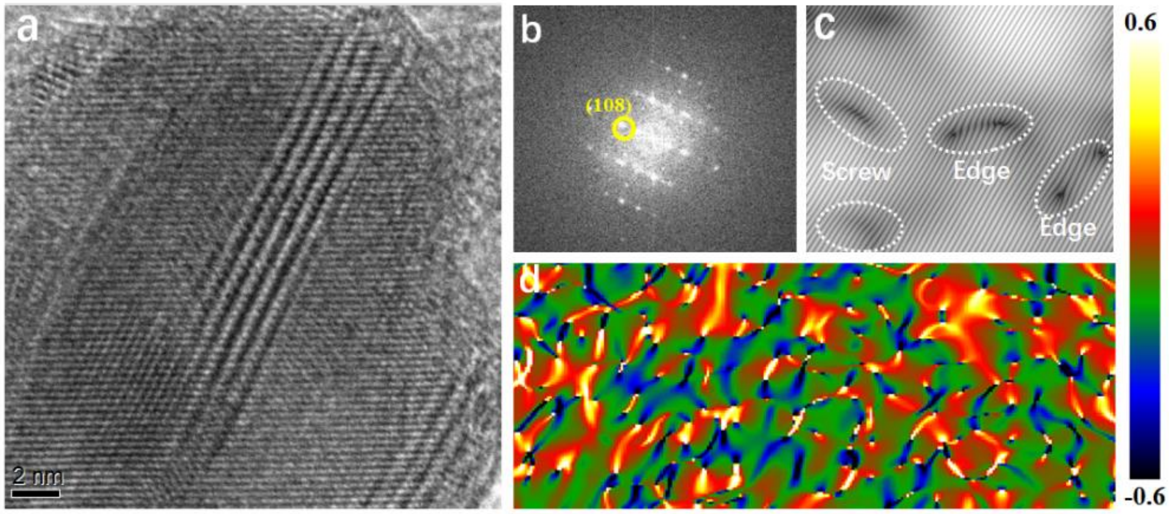

Fig. S12 a HRTEM image of the sodiation CuS electrode. **b, c** The corresponding FFT pattern and IFFT image based on (108) plane. **d** GPA strain distributions of (108) plane along ε_{xx}. (The color from green to dark blue represents the compressive strain, while the color from red to bright yellow represents the tensile strain). The GPA is realized by applying a plugin script installed in GATAN Digital Micrograph Software [S35]

The fast Fourier transform (FFT) patterns and inverse fast Fourier transform (IFFT) images of HRTEM lattice images were performed, as shown in Fig. S12a-c, respectively. That clearly reveals the formation of crystallographic defects of edge and screw dislocation after intercalating Na^+ ion, which inducing substantial strain into electrode materials. Figure 12d shows the GPA strain distributions of (108) plane along ε_{xx} (in-plane). The pink represents the maximum tensile strain, and the light green represents the maximum compressive strain. The evenly distributed color indicates the tensile and compressive stress is fairly uniform.

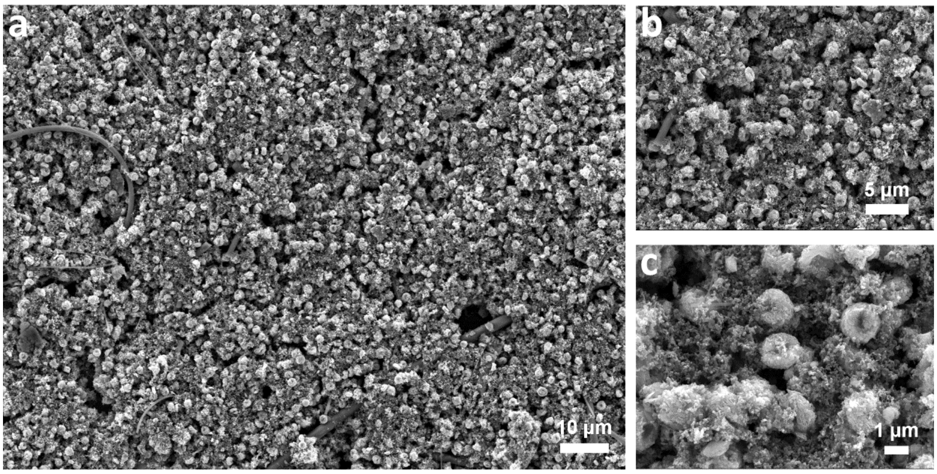

Fig. S13 SEM images of the CuS@PANI nanosheet arrays after 100 cycles

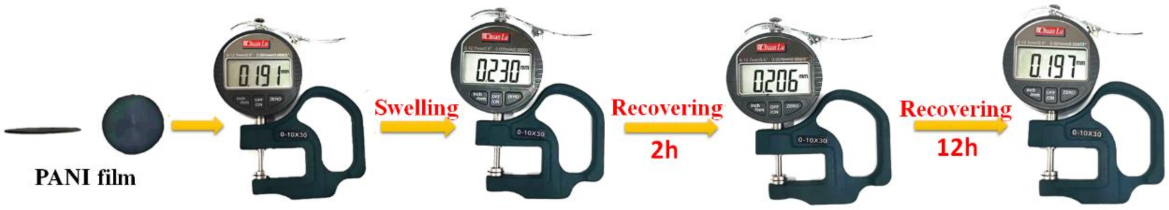

Fig. S14 The swelling experiment and the thickness changing of PANI were measured using a digital thickness gauge

The measurement results show the original PANI film with a thickness of 191 μm. After the PANI film was immersed in electrolyte for 3h, the thickness has been increased to 230 μm. Additionally, the swollen PANI were carried out a recovering experiment in vacuum drying environment. After 2 h, the thickness of PANI film back to 206 μm. With increasing drying time to 12 h, the PANI film returns to 197 μm, which is close to the original thickness of PANI

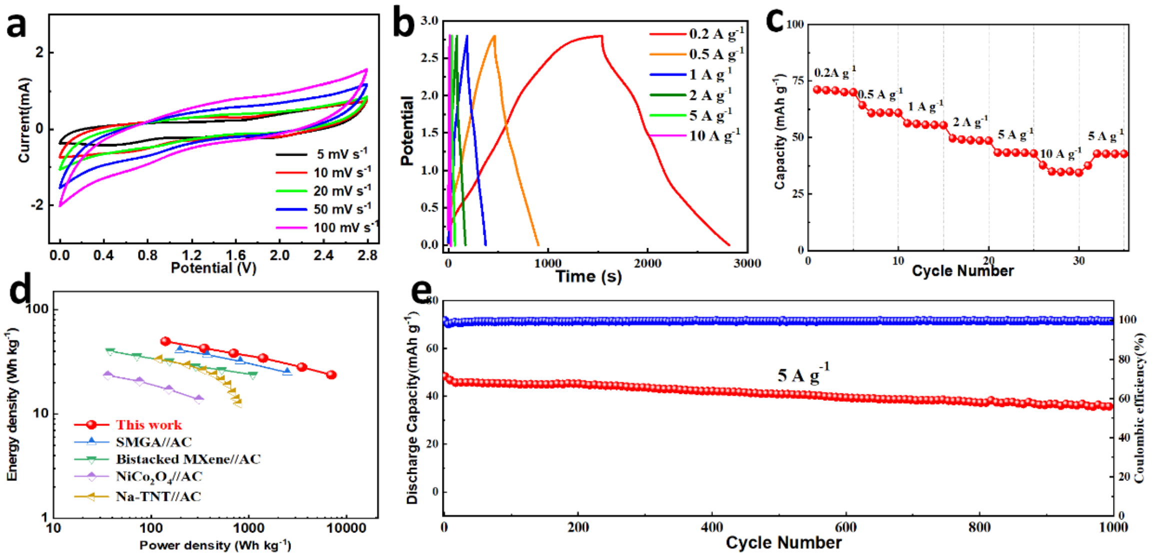

Fig. S15 (**a**) The CV curves of CuS@PANI//AC hybrid sodium-ion capacitors (SIC) at different current density. (**b**) The GCD curves and corresponding rate capacities of the SIC at rates ranging from 0.2 to 10 A g^{-1} (**c**). (**d**) Ragone plots of the CuS@PANI//AC compared to previously reported. (**e**) Cycling performance at a current density of 5 A g^{-1}

Figure S15a show the typical CV curves of CuS@PANI//AC SIC. The CV profile retained the quasi-rectangular shape at the scan rate from 5 to 100 mV s^{-1}, indicating high reversibility. Fig. S15b shows the nearly linear GCD curves of the CuS@PANI//AC SIC from 0.2 to 10 A g^{-1}. The corresponding rate capacities at different current densities are shown in Fig. S15c. The energy density (*E*) and power density (*P*) were calculated based on the following equations: $E = P \times t$, $P = \Delta V \times I/m$, and $\Delta V = (V_{max} + V_{min})/2$, where *t* is the discharge time (s), *I* is the discharge current (A), *m* is the mass of the active materials in both electrodes (*kg*), V_{max} is the voltage at the beginning of discharge after the *IR* drop, and V_{min} is the voltage at the end of discharge [S36]. As shown in Fig. S15d, the Ragone plot of CuS@PANI//AC present a high energy density of 50 Wh kg^{-1} at a power density of 140 W kg^{-1}, and ultrathin high-power output of 6250 W kg^{-1} at an impressive energy density of 23 Wh kg^{-1}. It can be clearly seen that the CuS@PANI//AC delivers an excellent rate performance, which can be attributed to the synergistic effect of the high rate pseudocapacitive process in CuS@PANI anode and fast electrical double layer capacitor (EDLC) in the AC cathode. The excellent energy/power density of CuS@PANI//AC are superior to previously reported sodium ion hybrid capacitors, such as SMGA (sulfur-doped $Ti_3C_2T_x$/RGO)//AC (41 Wh kg^{-1} at 197 W kg^{-1} and 25 Wh kg^{-1} at 2473 W kg^{-1}) [S37], Bi-stacked MXene//AC (40 Wh kg^{-1} at 38 W kg^{-1}) [S38], $NiCo_2O_4$//AC (23.5 Wh kg^{-1} at 36 W kg^{-1}) [S39], and Na-TNT (sodium titanate nanotubes)//AC (33.6 Wh kg^{-1} at 120 W kg^{-1}) [S40]. The cyclic performance of CuS@PANI//AC with a capacity retention of 98.7% for 200 cycles and 80.1% for 1000 cycles and high coulombic efficiency above 99.6% at 5 A g^{-1} demonstrates the competitive electrochemical stability of the SIC devices (as shown in Fig. S15e).

Supplementary References

[S1] Y. Li, Y. Liang, F.C.R. Hernandez, H.D. Yoo, Q. An et al., Enhancing sodium-ion battery performance with interlayer-expanded MoS_2–PEO nanocomposites. Nano Energy **15**, 453-461 (2015). https://doi.org/10.1016/j.nanoen.2015.05.012

[S2] B. Delley, From molecules to solids with the $DMol^3$ approach. J. Chem. Phys. **113**(18), 7756-7764 (2000). https://doi.org/10.1063/1.1316015

[S3] J.P. Perdew, K. Burke, M. Ernzerhof, Generalized gradient approximation made simple. Phys. Rev. Lett. **77**(18), 3865 (1996). https://doi.org/10.1103/PhysRevLett.77.3865

[S4] R. Sun, Q. Wei, Q. Li, W. Luo, Q. An et al., Vanadium sulfide on reduced graphene oxide layer as a promising anode for sodium ion battery. ACS Appl. Mater. Interfaces **7**(37), 20902-20908 (2015). https://doi.org/10.1021/acsami.5b06385

[S5] C. Zhu, P. Kopold, W. Li, P.A. Aken, J. Maier et al., A general strategy to fabricate carbon-coated 3D porous interconnected metal sulfides: case study of SnS/C nanocomposite for high-performance lithium and sodium ion batteries. Adv. Sci. **2**(12), 1500200 (2015). https://doi.org/10.1002/advs.201500200

[S6] W. Wang, L. Shi, D. Lan, Q. Li, Improving cycle stability of SnS anode for sodium-ion batteries by limiting Sn agglomeration, J. Power Sources **377**, 1-6 (2018). https://doi.org/10.1016/j.jpowsour.2017.11.084

[S7] S.H. Yang, S.K. Park, J.K. Kim, Y.C. Kang, A MOF-mediated strategy for constructing human backbone-like $CoMoS_3$@N-doped carbon nanostructures with multiple voids as a superior anode for sodium-ion batteries. J. Mater. Chem. A **7**(22), 13751-13761 (2019). https://doi.org/10.1039/C9TA03873F

[S8] Y. Liu, W. Zhong, C. Yang, Q. Pan, Y. Li et al., Direct synthesis of FeS/N-doped

carbon composite for high-performance sodium-ion batteries. J. Mater. Chem. A **6**(48), 24702-24708 (2018). https://doi.org/10.1039/C8TA08562E

[S9] F. Han, T. Lv, B. Sun, W. Tang, C. Zhang et al., In situ formation of ultrafine CoS_2 nanoparticles uniformly encapsulated in N/S-doped carbon polyhedron for advanced sodium-ion batteries. RSC Adv. **7**(49), 30699-30706 (2017). https://doi.org/10.1039/C7RA03628K

[S10] S. Liang, S. Zhang, Z. Liu, J. Feng, Z. Jiang et al., Approaching the theoretical sodium storage capacity and ultrahigh rate of layer-expanded MoS_2 by interfacial engineering on N-doped graphene. Adv. Energy Mater. **11**(12), 2002600 (2021). https://doi.org/10.1002/aenm.202002600

[S11] Y. Li, R. Zhang, W. Zhou, X. Wu, H. Zhang et al., Hierarchical MoS_2 hollow architectures with abundant mo vacancies for efficient sodium storage. ACS Nano **13**(5), 5533-5540 (2019). https://doi.org/10.1021/acsnano.9b00383

[S12] H. Shi, W. Lv, C. Zhang, D.W. Wang, G. Ling et al., Functional carbons remedy the shuttling of polysulfides in lithium–sulfur batteries: confining, trapping, blocking, and breaking up. Adv. Funct. Mater. **28**(38), 1800508 (2018). https://doi.org/10.1002/adfm.201800508

[S13] D. Sun, D. Ye, P. Liu, Y. Tang, J. Guo et al., MoS_2/graphene nanosheets from commercial bulky MoS_2 and graphite as anode materials for high rate sodium-ion batteries. Adv. Energy Mater. **8**(10), 1702383 (2017). https://doi.org/10.1002/aenm.201702383

[S14] X. Xu, R. Zhao, W. Ai, B. Chen, H. Du et al., Controllable design of MoS_2 nanosheets anchored on nitrogen-doped graphene: toward fast sodium storage by tunable pseudocapacitance. Adv. Mater. **30**(27), 1800658 (2018). https://doi.org/10.1002/adma.201800658

[S15] S. Zhou, S. Liu, W. Chen, Y. Cheng, J. Fan et al., A "biconcave-alleviated" strategy to construct aspergillus niger-derived carbon/MoS_2 for ultrastable sodium ion storage. ACS Nano **15**(8), 13814-13825 (2021). https://doi.org/10.1021/acsnano.1c05590

[S16] Z. Yuan, L. Wang, D. Li, J. Cao, W. Han, Carbon-reinforced Nb_2CTx MXene/MoS_2 nanosheets as a superior rate and high-capacity anode for sodium-ion batteries. ACS Nano **15**(4), 7439-7450 (2021). https://doi.org/10.1021/acsnano.1c00849

[S17] L. Tang, B. Zhang, T. Peng, Z. He, C. Yan et al., MoS_2/SnS@C hollow hierarchical nanotubes as superior performance anode for sodium-ion batteries. Nano Energy **90**, 106568 (2021). https://doi.org/10.1016/j.nanoen.2021.106568

[S18] H. He, X. Li, D. Huang, J. Luan, S. Liu, Electron-injection-engineering induced phase transition toward stabilized 1T-MoS_2 with extraordinary sodium storage performance. ACS Nano **15**(5), 8896-8906 (2021). https://doi.org/10.1021/acsnano.1c01518

[S19] S. Sui, H. Xie, M. Liang, B. Chen, C. Liu et al., "Three-in-one" multi-level design of MoS_2-based anodes for enhanced sodium storage: from atomic to macroscopic level. Adv. Funct. Mater. **32**(16), 2110853 (2022). https://doi.org/10.1002/adfm.202110853

[S20] X. Hu, Y. Liu, J. Li, G. Wang, J. Chen et al., Self-assembling of conductive interlayer-expanded WS_2 nanosheets into 3D hollow hierarchical microflower bud hybrids for fast and stable sodium storage. Adv. Funct. Mater. **30**(5), 1907677 (2019). https://doi.org/10.1002/adfm.201907677

[S21] Y. Wang, D. Kong, S. Huang, Y. Shi, M. Ding et al., 3D carbon foam-supported WS_2 nanosheets for cable-shaped flexible sodium ion batteries. J. Mater. Chem. A **6**(23),

10813-10824 (2018). https://doi.org/10.1039/C8TA02773K

[S22] Y. Wang, D. Kong, W. Shi, B. Liu, G.J. Sim et al., Ice templated free-standing hierarchically WS_2/CNT-rGO aerogel for high-performance rechargeable lithium and sodium ion batteries. Adv. Energy Mater. **6**(21), 1601057 (2016). https://doi.org/10.1002/aenm.201601057

[S23] X. Wang, J. Huang, J. Li, L. Cao, W. Hao et al., Improved Na storage performance with the involvement of nitrogen-doped conductive carbon into WS_2 nanosheets. ACS Appl. Mater. Interfaces **8**(36), 23899-23908 (2016). https://doi.org/10.1021/acsami.6b06032

[S24] Y.V. Lim, Y. Wang, D. Kong, L. Guo, J.I. Wong et al., Cubic-shaped WS_2 nanopetals on a prussian blue derived nitrogen-doped carbon nanoporous framework for high performance sodium-ion batteries. J. Mater. Chem. A **5**(21), 10406-10415 (2017). https://doi.org/10.1039/C7TA01821E

[S25] P.A. Aken, Engineering nanostructured electrode materials for high performance sodium ion batteries: a case study of a 3D porous interconnected WS_2/C nanocomposite. J. Mater. Chem. A **3**(41), 20487-20493 (2015). https://doi.org/10.1039/C5TA05758B

[S26] D. Su, S. Dou, G. Wang, WS_2@ graphene nanocomposites as anode materials for Na-ion batteries with enhanced electrochemical performances. Chem. Commun. **50**(32), 4192-4195 (2014). https://doi.org/10.1039/C4CC00840E

[S27] S.H. Choi, Y.C. Kang, Sodium ion storage properties of WS_2-decorated three-dimensional reduced graphene oxide microspheres. Nanoscale **7**(9), 3965-3970 (2015).

[S28] X. Hu, Q. Peng, T. Zeng, B. Shang, X. Jiao et al., Promotional role of nano TiO_2 for pomegranate-like SnS_2@ C spheres toward enhanced sodium ion storage. Chem. Eng. J. **363**, 213-223 (2019). https://doi.org/10.1016/j.cej.2019.01.130

[S29] J. Wang, C. Luo, J. Mao, Y. Zhu, X. Fan et al., Solid-state fabrication of SnS_2/C nanospheres for high-performance sodium ion battery anode, ACS Appl. Mater. Interfaces **7**(21), 11476-11481 (2015). https://doi.org/10.1021/acsami.5b02413

[S30] W. Sun, X. Rui, D. Yang, Z. Sun, B. Li et al., Two-dimensional tin disulfide nanosheets for enhanced sodium storage. ACS Nano **9**(11), 11371-11381 (2015). https://doi.org/10.1021/acsnano.5b05229

[S31] Y. Zhang, P. Zhu, L. Huang, J. Xie, S. Zhang et al., Few-layered SnS_2 on few-layered reduced graphene oxide as Na-ion battery anode with ultralong cycle life and superior rate capability. Adv. Funct. Mater. **25**(3), 481-489 (2015). https://doi.org/10.1002/adfm.201402833

[S32] P. Kumar, M. Gusain, R. Nagarajan, Synthesis of $Cu_{1.8}S$ and CuS from copper-thiourea containing precursors; anionic (Cl^-, NO^{3-}, SO_4^{2-}) influence on the product stoichiometry. Inorg. Chem. **50**(7), 3065-3070 (2011). https://doi.org/10.1021/ic102593h

[S33] M. Gabal, M. Hussein, A. Hermas, Synthesis, characterization and electrical conductivity of polyaniline-$Mn_{0.8}Zn_{0.2}Fe_2O_4$ nano-composites, Int. J. Electrochem. Sci **11**, 4526-4538 (2016). https://doi.org/10.20964/2016.06.20

[S34] Y. Jiang, X. Zhang, Q.Q. Ge, B.B. Yu, Y.G. Zou et al., ITO@Cu_2S tunnel junction nanowire arrays as efficient counter electrode for quantum-dot-sensitized solar cells. Nano Lett. **14**(1), 365-372 (2014). https://doi.org/10.1021/nl404251p

[S35] S. Liu, Z. Hu, Y. Wu, J. Zhang, Y. Zhang et al., Dislocation-strained IrNi alloy nanoparticles driven by thermal shock for the hydrogen evolution reaction. Adv. Mater. **32**(48), 2006034 (2020). https://doi.org/10.1002/adma.202006034

[S36] R. Jia, Y. Jiang, R. Li, R. Chai, Z. Lou et al., Nb_2O_5 nanotubes on carbon cloth for high performance sodium-ion capacitors. Sci. China Mater. **63**(7), 1171-1181 (2020). https://doi.org/10.1007/s40843-020-1278-9

[S37] F. Song, J. Hu, G. Li, J. Wang, S. Chen et al., Room-temperature assembled MXene-based aerogels for high mass-loading sodium-ion storage. Nano-Micro Lett. **14**, 37 (2021). https://doi.org/10.1007/s40820-021-00781-6

[S38] N. Kurra, M. Alhabeb, K. Maleski, C.H. Wang, H.N. Alshareef et al., Bistacked titanium carbide (MXene) anodes for hybrid sodium-ion capacitors. ACS Energy Lett. **3**(9), 2094-2100 (2018). https://doi.org/10.1021/acsenergylett.8b01062

[S39] R. Ding, L. Qi, H. Wang, An investigation of spinel $NiCo_2O_4$ as anode for Na-ion capacitors. Electrochim. Acta **114**, 726-735 (2013). https://doi.org/10.1016/j.electacta.2013.10.113

[S40] J. Yin, L. Qi, H. Wang, Sodium titanate nanotubes as negative electrode materials for sodium-ion capacitors. ACS Appl. Mater. Interfaces **4**(5), 2762-2768 (2012). https://doi.org/10.1021/am300385r
